# Mapping general anesthesia states based on electro-encephalogram transition phases

**DOI:** 10.1016/j.neuroimage.2023.120498

**Published:** 2024-01

**Authors:** V. Loison, Y. Voskobiynyk, B. Lindquist, D. Necula, D. Longrois, J. Paz, D. Holcman

**Affiliations:** a Group of Data Modeling and Computational Biology, Institut de Biologie (IBENS), École Normale Supérieure CNRS, Université PSL Paris, France; b Gladstone Institutes, USA; c Gladstone Institute of Neurological Disease, University of California, San Francisco, USA; d Département d’Anesthésie-Réanimation, Hôpital Bichat-Claude Bernard, Assistance Publique-Hôpitaux de Paris, Paris, France; e DAMPT, University of Cambridge and Churchill College, CB30DS, Cambridge, UK

**Keywords:** Electro-encephalography, General Anesthesia, Isoflurane, Iso-electric suppression, State chart, Spectral decomposition, IRASA, Machine Learning, Classification

## Abstract

Cortical electro-encephalography (EEG) served as the clinical reference for monitoring unconsciousness during general anesthesia. The existing EEG-based monitors classified general anesthesia states as underdosed, adequate, or overdosed, lacking predictive power due to the absence of transition phases among these states. In response to this limitation, we undertook an analysis of the EEG signal during isoflurane-induced general anesthesia in mice. Adopting a data-driven approach, we applied signal processing techniques to track θ- and δ-band dynamics, along with iso-electric suppressions. Combining this approach with machine learning, we successfully developed an automated algorithm. The findings of our study revealed that the dampening of the δ-band occurred several minutes before the onset of significant iso-electric suppression episodes. Furthermore, a distinct γ-frequency oscillation was observed, persisting for several minutes during the recovery phase subsequent to isoflurane-induced overdose. As a result of our research, we generated a map summarizing multiple brain states and their transitions, offering a tool for predicting and preventing overdose during general anesthesia. The transition phases identified, along with the developed algorithm, have the potential to be generalized, enabling clinicians to prevent inadequate anesthesia and, consequently, tailor anesthetic regimens to individual patients.

## Highlights

• EEG allows estimating the depth of general anesthesia (GA).
• Segmentation and spectral methods to classify GA states as “adequate”/underdosed/overdosed where transitions among these states are estimated.
• Predictive map represents gradual sedation states during GA.
• Gamma rebound as an *a posteriori* marker of isoflurane overdose.
• New paradigm for individually tailored and predictive anesthetic regimens.

## Graphical abstract

**Figure:**
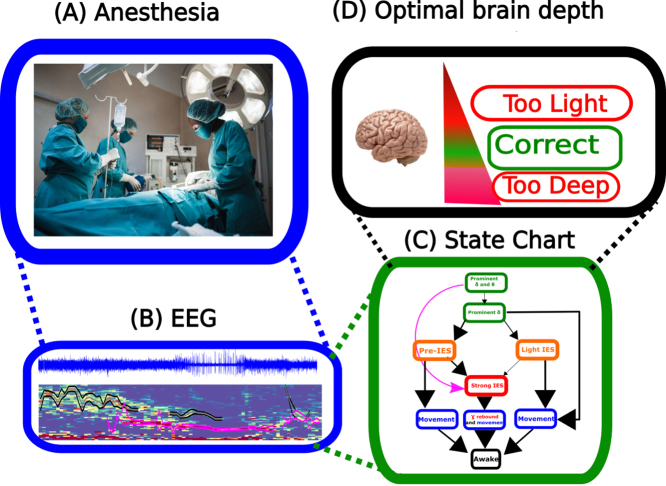

## 1 Introduction

Over the past century, the analysis of cortical electroencephalogram (EEG) data has led to comprehensive classifications of brain states. In the last five decades, advancements in spectral analysis and signal segmentation of EEG data have matured, offering valuable insights into the instantaneous dynamics of brain activity during sleep (Gorgoni et al., 2020), coma (Maas et al., 2017, André-Obadia et al., 2018), and general anesthesia (GA) (Purdon et al., 2015, Constant and Sabourdin, 2012). EEG is now a routine tool for monitoring the adequacy, or depth, of anesthesia in humans.

While recent data has highlighted associations between anesthetic overdose and post-operative complications (Soehle et al., 2015, Fritz et al., 2016a), the impact of real-time monitoring overdose alerts provided to anesthesiologists on improving outcomes remains controversial (Wildes et al., 2019, Avidan et al., 2008, Whitlock et al., 2011). These observations underscore the need for preventing hypnotic overdose to enhance outcomes. This prevention, rather than correction after the fact, necessitates a new paradigm for EEG analysis.

During anesthesia with agents like propofol or halogenated gases such as sevoflurane or isoflurane, the brain undergoes transitions characterized by the presence of frontal α-oscillations in the 8–12 Hz range (Buzsáki, 2006). Increasing hypnotic concentration can lead to the disappearance of α-oscillations, resulting in partial suppressions of the α-band known as α-suppressions (αS) (Cartailler et al., 2019, Sun and Holcman, 2022). In contrast, in rodents, general anesthesia (GA) is characterized by the presence of θ- and δ-oscillations (Guidera et al., 2017). Further increases in hypnotic concentration in both humans and rodents can result in iso-electric suppressions (IES), representing profound anesthesia and being associated with post-anesthetic complications such as delirium and cognitive dysfunction in humans (Fritz et al., 2016b, Soehle et al., 2015-04-28).

Post-operative delirium has also been observed in mice (Peng et al., 2016), but its connection to IES is not thoroughly investigated. Temporal relationships have been established between EEG patterns in humans, where α-band suppressions precede IES appearance in patients anesthetized with propofol (Cartailler et al., 2019). This allows for the prediction of patients most sensitive to overdose within the first 10 min of GA with propofol (Sun and Holcman, 2022). However, a similar approach has not been developed for GA induced by halogenated gases, given the different EEG signatures they produce (Kenny et al., 2014). Thus, our investigation focused on identifying temporal relationships between EEG patterns in isoflurane-induced GA.

Various computational methods are employed for EEG analysis, including wavelets (Jaffard et al., 2001, Worrell et al., 2012), thresholding methods (Donoho and Johnstone, 1994), and empirical mode decomposition (Ho and Hung, 2020). Separating oscillatory components from spectral decay provides valuable insights into neurophysiological signals (Wen and Liu, 2016, Donoghue et al., 2020, Ouyang et al., 2020), especially for EEG recordings during general anesthesia (Brake et al., 2021). Based on EEG and electromyogram (EMG) recordings during isoflurane-induced GA in mice, we developed here a signal-processing approach coupled with machine learning. This integration allowed us to identify relevant patterns and assess their predictive power. Our EEG time-frequency analysis relies on irregular sampling auto-spectral analysis (IRASA) (Wen and Liu, 2016), effectively isolating oscillatory components from background spectral decay. By computing the relative power of frequency bands and the time proportion of IES, we uncovered multiple EEG and EMG states, revealing specific robust transitions between them. This led us to propose a state chart representing brain states and their associated transitions, which could be utilized to assess and predict the depth of anesthesia in isoflurane-induced GA.

**Fig. 1:**
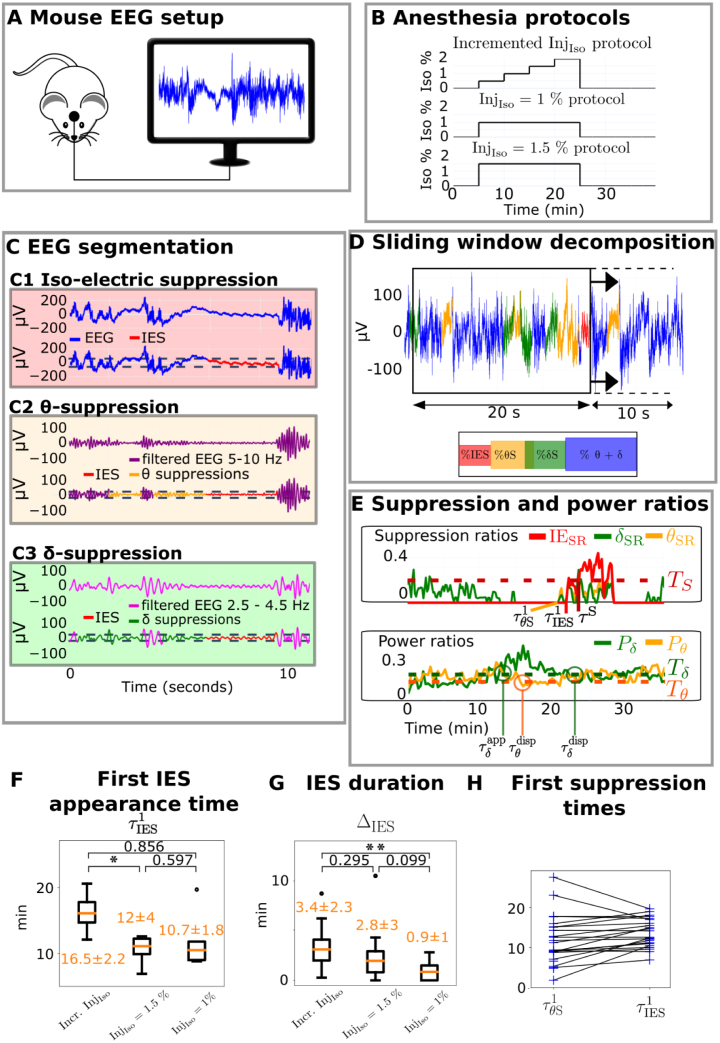
**Time-frequency segmentation of EEG recorded during isoflurane-induced GA in mice. (A)** Experimental setting for a single-electrode EEG recording. **(B)** Three protocols used for isoflurane-induced GA. **(C)** Suppression detection leading to **(C1)** IES (red), **(C2)**θ-suppression (yellow), **(C3)**δ-suppression (green). Segments where the signal is lower than a threshold (dotted lines) are labeled as suppressions (see Methods). **(D)** Time spent in each suppression type in a sliding time window. **(E)** Suppression ratios across the EEG recording, with the delay to first IES $\tau_{\text{IES}}^{1}$, the IES ratio exceeds threshold $T_{S}$ at time $\tau^{S}$, and relative power of the θ and δ rhythms. **(F)** Distribution of delay from GA induction to first IES $\tau_{\text{IES}}^{1}$. **(G)** Distribution of cumulative time spent in IES $\Delta_{\text{IES}}$. **(H)** Delay from GA induction to the first θ-suppression occurrence, and first IES occurrence respectively. The black lines link two points coming from the same recording. *P<0.05, **P<0.001, two-sided Wilcoxon-rank U test.

## 2 Methods

### 2.1 Animal care and ethics statement

All protocols were approved by the Institutional Animal Care and Use Committee at the University of California, San Francisco, and Gladstone Institutes, with institutional oversight. Experiments were conducted according to ARRIVE guidelines (Kilkenny et al., 2012) and recommendations to facilitate transparent reporting (Landis et al., 2012). Experiments were approved by the Institutional Animal Care and Use Committee under IACUC protocol number AN189059-02N. The present experiments were performed in a AAALAC-accredited facility. All biological variables were documented. Adult C57BL/6J mice were used for each experiment. Mice of both sexes were used for the current study. Precautions were taken to minimize distress and the number of animals used in each set of experiments. Mice were housed in a pathogen-free barrier facility on a standard 12-h light/dark cycle with ad libitum access to food and water.

### 2.2 Surgical implantation of the EEG and EMG devices and EEG recordings

Seventeen adult mice underwent surgical implantation of EMG and EEG devices for chronic electromyogram and electrocorticogram recordings. Mice were anesthetized with vaporized isoflurane (3% induction, 1−2% maintenance, carried by 100% O${}_{2}$ at a flow rate of 2 L/min) and placed under a stereotaxic frame for chronic EEG implants as previously described in Holden et al. (2021) and Cho et al. (2022). Briefly, an EEG screw was implanted in the skull overlying the cortical region at the following coordinates: 1.0 mm anterior from Bregma and 2.5 mm lateral from the midline (Kirkcaldie et al., 2012). A ground screw was placed overlying the cerebellum (0.5–1 mm posterior to Lambda and 0.5–1 mm lateral to midline). The coordinates are computed from the primary somatosensory region (S1). The EMG electrode was placed in the deep parasagittal cervical muscles. The skin was closed over the entire apparatus, which was sealed with dental cement and Vetbond tissue adhesive. For analgesia, topical lidocaine ointment (5%) was applied prior to incision and extended-release buprenorphine (0.05–0.1 mg/kg s.c.) was administered prior to recovery from anesthesia. Mice recovered for 5–7 days after surgery before the start of recordings.

Two types of EEG devices were used: purchased wireless telemetry devices (HD-X02, Data Sciences International (DSI), St. Paul, MN) and custom-made wired EEG devices made in the Paz lab using cortical screws connected to a Millmax device (Cho et al., 2022). Wireless recordings were acquired using Ponemah software (DSI); wired recordings were acquired using Synapse software (Tucker Davis Technologies).

### 2.3 Protocols using isoflurane-induced GA

GA was induced in the mobile isoflurane anesthesia induction chambers. Mice were placed into the plastic chambers, and EEG was recorded for 5 min before vaporized isoflurane carried by 100% O2 at a flow rate of 2L/min was turned on. There were three different protocols for isoflurane-induced GA. In the first, the isoflurane dose was gradually increased from 0.5% to 2% every 5 min with 0.5% increments (n=14), while in the other two, the dose was fixed at 1% (n=10) and 1.5% (n=9) (Fig. 1A–B). Vaporized isoflurane was turned off after 20 min. Mice were kept in the plastic induction chamber until full recovery and visible unimpaired movement around the plastic chamber, usually no more than 20 min. Mice were returned to home cages at the end of the experiment. These protocols were chosen in order to characterize and compare the mouse EEG response to isoflurane under constant light sedation (1% protocol), constant high sedation (1.5% protocol), and increasing concentration from light to high sedation (incremented protocol). Due to experimental constraints, some mice were used for several protocols, with at least one resting day between two sessions. Eight mice underwent the incremented and 1.5% protocol, four mice underwent the step protocol alone, three mice underwent the 1% protocol, and two mice underwent the 1% protocol three time, the 1.5% protocol once, and the incremented protocol once. Although we observed significant intra-individual variability in responses to isoflurane, we did not observe group differences to suggest a major confounding effect of repeated isoflurane exposure (Supplementary section S1.7).

### 2.4 EEG data and pre-processing

The EEG signal S(t) was digitized at a sampling frequency $f_{s}=500\;\mathrm{Hz}$. We first identified artifacts, like regions where the signal is constant and equal to 0 due to no signal being recorded. Regions with abnormally high values were also identified as artifacts using hysteresis thresholding with a low threshold of 0.08 μV and a high threshold of 1200 μV (Canny, 1986). We labeled the artifact regions as NaN (not containing any significant signal to be processed). Three EEG recordings (one per anesthesia protocol) were excluded because they contained too many artifacts.

### 2.5 Signal processing tools

Signal processing notations used throughout the Methods section are defined here. For some computations specified below, the signal S was band-pass filtered using a Butterworth forward–backward filter (Butterworth, 1930) of order 1 (effective order 4) in a frequency domain [$f_{1}$, $f_{2}$], where the frequencies $f_{1}$ and $f_{2}$ are specified in Table 1. A sliding window $W_{w}\left(t\right)$ centered at time t and of width w was used to compute statistical markers locally in time. For $w\in R^{+}$ and $t>\frac{w}{2}$, we use the notation

(1) $$W_{w}\left(t\right)=\left[t-\frac{w}{2},t+\frac{w}{2}\right]$$

We used an overlap between two sliding windows of $\frac{w}{2}$ or 0. Chosen values of w and overlap are specified in Table 1.

#### 2.5.1 EEG segmentation of suppression periods

To detect IES, regions where the amplitude of the EEG was smaller than the threshold $T_{\text{IES}}$ for at least 1 s were identified. The threshold chosen was

$$T_{\text{IES}}=r_{\text{IES}}{\text{RMS}}_{\text{EEG}},$$

where $r_{\text{IES}}=0.7$ and ${\text{RMS}}_{\text{EEG}}$ is the Root Mean Square of the entire recording:

$${\text{RMS}}_{\text{EEG}}=\sqrt{\frac{1}{T}\int_{0}^{T}S^{2}\left(t\right)\text{d}t}.$$

The threshold was chosen using a novel heuristic, see supplementary section 2.1, Fig. S1, and Fig. S2. Two detected suppressions separated by less than 0.5 s were merged as one single suppression (Cartailler et al., 2019). The detection of IES is shown in Fig. 1C1.

Similarly, suppressions of the θ- (resp. δ-) rhythm were identified (Cartailler et al., 2019) directly on the raw EEG signal, without the spectral decomposition (Section 2.7). The dynamics of the δ− and θ− bands were computed directly on the EEG signal, without using the IRASA decomposition. Similarly for the suppression episodes that were segmented directly on the EEG, without using the IRASA spectral decomposition. In brief, the signal was first band-pass filtered within the 5−10 Hz (resp. 2.5−4.5 Hz) band. Suppression segments where the amplitude of the filtered signal was below the threshold $T_{\theta \text{S}}=0.2\;{\text{RMS}}_{\text{EEG}}$ (resp. $T_{\delta \text{S}}=0.2\;{\text{RMS}}_{\text{EEG}}\text{)}$ were labeled as θ- (resp. δ-) suppressions (Fig. 1C2–3). Although the value T=0.2 for the suppression threshold was chosen empirically, this value is comparable to the one chosen in Sun and Holcman (2022) and Cartailler et al. (2019). We further tested several values and retain the one that best detected the band suppression, limiting false detection of non-suppression episodes. Because IES can also be detected as θ− and δ− suppressions, segments detected as IES were removed from θ and δ suppressions. A general principle to assign optimal value to threshold remains an open question.

**Table 1:** Main parameters.

Parameter	Symbol	Value
Sampling frequency (Hz)	$f_{s}$	500Hz

Root Mean Square of the EEG	${\text{RMS}}_{\text{EEG}}$	
Relative IES threshold	$r_{\text{IES}}$	0.7
Threshold for IES detection	$T_{\text{IES}}=r_{\text{IES}}{\text{RMS}}_{\text{EEG}}$	
Threshold for θ suppressions detection	$T_{\theta \text{S}}=0.2{\text{RMS}}_{\text{EEG}}$	
Threshold for δ suppressions detection	$T_{\delta \text{S}}=0.2{\text{RMS}}_{\text{EEG}}$	
Suppression ratios time window width	20 s	
Suppression ratios time window overlap	10 s	
IES threshold	$T_{S}$	0.25

Power ratio time window width		20 s
Power ratio time window overlap		0 s
Threshold for δ relative power	$T_{\delta }$	0.15
Threshold for θ relative power	$T_{\theta }$	0.1
Power ratio of the δ-band	$P_{\delta }\left(t\right)$	
Power ratio of the θ-band	$P_{\theta }\left(t\right)$	

Spectral time window width	$W_{t}$	60 s
Spectral time window overlap		30 s
Coefficients of the 1/f-fit	$a_{t},c_{t}$	
Exponent of the 1/f-fit	$p_{t}$	
Rhythm amplitude of the δ band	$b_{\delta }\left(t\right)$	
Center frequency	$f_{\delta }\left(t\right)$	
standard deviation	$\sigma_{\delta \left(t\right)}$	

Rhythm amplitude of the θ band	$b_{\theta }\left(t\right)$	
Center frequency	$f_{\theta }\left(t\right)$	
Standard deviation	$\sigma_{\theta \left(t\right)}$	

#### 2.5.2 Estimating suppression ratios

We define the iso-electric suppression Ratio (${\text{IES}}_{\text{SR}}$) as the proportion of time that the EEG signal spends in IES inside a sliding window $W_{R}\left(t\right)$ of width R (Eq. (1)), that is

(2) $${\text{IES}}_{\text{SR}}\left(t\right)=\frac{\text{Duration of IES in}W_{R}\left(t\right)}{R}.$$

Similarly, we define the θ-Suppression Ratio $\theta_{\text{SR}}$ as

(3) $$\theta_{\text{SR}}\left(t\right)=\frac{\text{Duration of}\theta \;\text{suppressions in}W_{R}\left(t\right)}{R}.$$

Finally, the δ-Suppression Ratio is

(4) $$\delta_{\text{SR}}\left(t\right)=\frac{\text{Duration of}\delta \;\text{suppressions in}W_{R}\left(t\right)}{R},$$

These suppression ratios are computed on sliding time windows of width R=20 s and an overlap of 10 s (Fig. 1E). Notably, the iso-electric suppression ratio is very similar to the commonly used burst suppression ratio (Rampil et al., 1988).

#### 2.5.3 Detecting strong IES episodes

Segments where the IES ratio (Eq. (2)) was high were collected. IES at time t is considered strong if more than a threshold $T_{S}=25\text{\%}$ of the time window centered in t is detected as IES:

(5) $${\text{IES}}_{\text{SR}}\left(t\right)>T_{S}.$$

This threshold was chosen empirically after a visual inspection of EEG.

Therefore, the first strong IES time $\tau^{\text{S}}$ is the first time at which ${\text{IES}}_{\text{SR}}\left(t\right)>T_{S}$ on consecutive time windows for at least 40 s.

(6) $$\tau^{\text{S}}=\underset{t\in \left[\tau_{\text{Iso}}^{\text{start}},\tau_{\text{rec}}^{\text{stop}}\right]}{\min}\left\{t-\tau_{\text{Iso}}^{\text{start}}\;|\;\text{for all}\;t^{\prime }\in \left[t,t+40s\right],{\text{IES}}_{\text{SR}}\left(t^{\prime }\right)>T_{\text{S}}\right\},$$

where $\tau_{\text{Iso}}^{\text{start}}$ is the beginning of induction time, and $\tau_{\text{rec}}^{\text{stop}}$ is the end of recording time.

### 2.6 Computing frequency power ratios from EEG and detecting present frequency rhythms

#### 2.6.1 Computing power ratios of the δ- and θ-bands

The power of the low-pass filtered EEG $S_{20}$ under 20 Hz, the band-pass filtered signal $S_{\theta }$ in 5−10 Hz, and $S_{\delta }$ band-pass filtered in 2.5−4.5 Hz were computed. $S_{20}$, $S_{\theta }$ and $S_{\delta }$ are taken directly from the EEG, without the spectral decomposition developed in Section 2.7. The associated powers are computed in the sliding time window $W_{R}\left(t\right)$:

$$p_{\text{EEG20}}\left(t\right)=\frac{1}{R}\int_{W_{R}\left(t\right)}S_{20}^{2}\left(s\right)\text{d}s$$

$$p_{\theta }\left(t\right)=\frac{1}{R}\int_{W_{R}\left(t\right)}S_{\theta }^{2}\left(s\right)\text{d}s$$

$$p_{\delta }\left(t\right)=\frac{1}{R}\int_{W_{R}\left(t\right)}S_{\delta }^{2}\left(s\right)\text{d}s,$$

The θ- and δ- power ratios are defined by

(7) $$P_{\theta }\left(t\right)=\frac{p_{\theta }\left(t\right)}{p_{\text{EEG20}}\left(t\right)},\;P_{\delta }\left(t\right)=\frac{p_{\delta }\left(t\right)}{p_{\text{EEG20}}\left(t\right)}$$

and computed over a sliding windows $W_{R}\left(t\right)$ with R=20 s and no overlap (Eq. (1), Fig. 1E).

#### 2.6.2 Activity of frequency bands

To assess whether the θ- or δ- rhythm is prominent, its power ratio is computed (Eq. (7)) over the sliding windows defined above (Eq. (1)) and compare it to a threshold $T_{\theta }=0.1$ or $T_{\delta }=0.15$. The θ rhythm (respectively δ rhythm) is considered to be prominent at time t if $P_{\theta }>T_{\theta }$ (respectively $P_{\delta }>T_{\delta }$) for at least 1 min. Conversely, the dampening time of the θ rhythm (respectively δ rhythm) is defined as when $P_{\theta }<T_{\theta }$ (respectively $P_{\delta }<T_{\delta }$) for at least 1 min. For instance in Fig. 1E, the δ rhythm is absent during the 0−11 min period, prominent during the 11−22 min period, dampened during the 22−27 min period, and prominent during the 27−33 min period. The first time of δ appearance $\tau_{\delta }^{\text{app}}$ is defined as the first time at which the δ rhythm is prominent since the beginning of anesthesia:

(8) $$\tau_{\delta }^{\text{app}}=\underset{t\in \left[\tau_{\text{Iso}}^{\text{start}},\tau_{\text{rec}}^{\text{stop}}\right]}{\min}\left\{t-\tau_{\text{Iso}}^{\text{start}}\;|\;\text{for all}\;t^{\prime }\in \left[t,t+1\text{min}\right],P_{\delta }\left(t^{\prime }\right)>T_{\delta }\right\},$$

where $\tau_{\text{Iso}}^{\text{start}}$ is the beginning of induction time and $\tau_{\text{rec}}^{\text{stop}}$ is the end time of the recording. The first time of θ appearance is not defined, because this rhythm is already prominent before the beginning of anesthesia. Similarly, the time of dampening of the θ and δ rhythm are defined as follows:

(9) $$\tau_{\theta }^{\text{disp}}=\underset{t\in \left[\tau_{\text{Iso}}^{\text{start}},\tau_{\text{rec}}^{\text{stop}}\right]}{\min}\left\{t-\tau_{\text{Iso}}^{\text{start}}\;|\;\text{for all}\;t^{\prime }\in \left[t,t+1\text{min}\right],P_{\theta }\left(t^{\prime }\right)<T_{\theta }\right\},$$

(10) $$\tau_{\delta }^{\text{disp}}=\underset{t\in \left[\tau_{\delta }^{\text{app}},\tau_{\text{rec}}^{\text{stop}}\right]}{\min}\left\{t-\tau_{\text{Iso}}^{\text{start}}\;|\;\text{for all}\;t^{\prime }\in \left[t,t+1\text{min}\right],P_{\delta }\left(t^{\prime }\right)<T_{\delta }\right\}.$$

### 2.7 Extracting dominant frequency rhythms present in the EEG

To quantify the persistence of a frequency rhythm present in the EEG signal,an algorithm (Wen and Liu, 2016) which separates the power spectrum into 1/f and oscillatory components was adapted. The novelty consists in using this decomposition in sliding time windows, which results in a decomposition that is continuous over time.

**Fig. 2:**
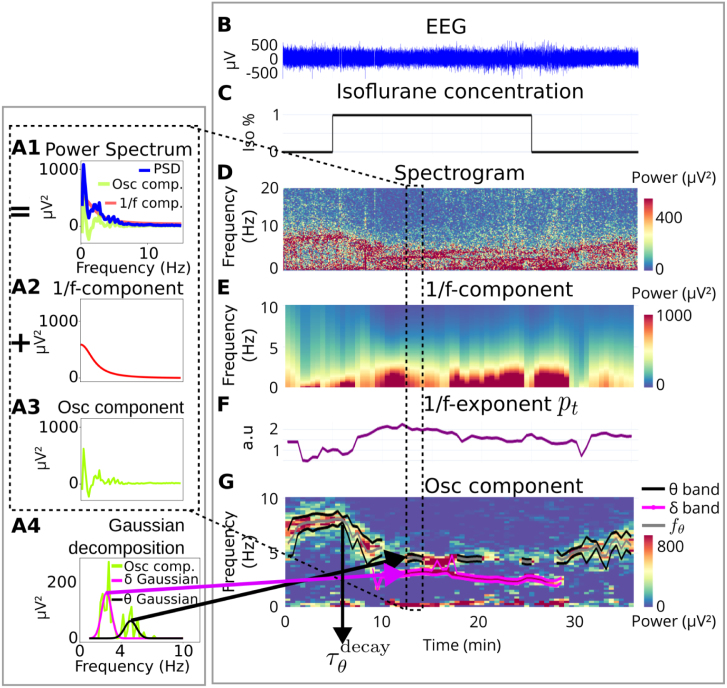
**EEG Spectral analysis applied to isoflurane-induced GA in mice** on a single one-minute time window **(A)**, and along the entire procedure **(B–G)**. The Power Spectral Density (PSD) of one minute of EEG is computed **(A1)**, and separated into the 1/f-component **(A2)** and the oscillatory component **(A3)** (Eq. (12)). The θ- (pink) and δ- (black) band characteristics of this time window are obtained by fitting Gaussians to the oscillatory component **(A4)**. This separation is performed on successive time windows along the procedure, resulting in a continuous estimation of the spectral parameters. **(B)** EEG recording on which the estimation is applied. **(C)** Anesthesia protocol. **(D)** Spectrogram of the entire EEG recording. **(E)** Estimated 1/f component. **(F)** Exponent of 1/f component. **(G)** Estimated oscillatory component and tracked θ and δ rhythms.

#### 2.7.1 Spectral decomposition on a sliding time window

The power spectral density ${\text{PSD}}_{t,w}\left(f\right)$ of the signal S is computed over the time window $W_{w}\left(t\right)$ (Eq. (1)) and can be decomposed as follows:

$${\text{PSD}}_{t,w}\left(f\right)={\text{1/f}}_{t,w}\left(f\right)+{\text{Osc}}_{t,w}\left(f\right),\text{where}$$

(11) $${\text{1/f}}_{t,w}\left(f\right)=\frac{a_{t}}{c_{t}+f^{p_{t}}},\text{and}$$

$${\text{Osc}}_{t,w}\left(f\right)=\underset{1\leq k_{t}\leq N_{t}}{\sum }b_{k_{t}}\exp\left(-\frac{{\left(f-f_{k_{t}}\right)}^{2}}{2\sigma_{k_{t}}^{2}}\right),$$

see Fig. 2A and Buzsáki (2006). The first component $1/f_{t,w}$ captures the frequency decay of the power spectral density and is characterized by the amplitude $a_{t}$, the exponent $p_{t}$ and the correction term $c_{t}$, which we estimate, as discussed below. We added a correction term $c_{t}$ in the denominator of the 1/f-model, leading to the term $\frac{a_{t}}{c_{t}+f^{p_{t}}}$, which now converges to a finite value when the frequency f tends to zero. This correction term allows us to fit the 1/f-component until the origin and accounts for the finite power spectral densities of the present EEG recordings. Second, introducing the correction term $c_{t}$ divides by 15 the approximation error, see supplementary section S2.2, Fig. S3 and Fig. S4. The second component ${\text{Osc}}_{t,w}$ accounts for the oscillatory part of the signal and can be decomposed as a sum of $N_{t}$ Gaussians (Fig. 2A3–4). Each Gaussian peaks at the main frequency $f_{k_{t}}$ with a standard deviation $\sigma_{k_{t}}$ and amplitude $b_{k_{t}}$. The oscillatory parameters to be estimated are the number $N_{t}$ and the Gaussian parameters ${\left\{b_{k_{t}},f_{k_{t}},\sigma_{k_{t}}\right\}}_{1\leq k_{t}\leq N_{t}}$. In practice, there are at most two main components in the 0−20 Hz domain: the θ and δ bands, so that

(12) $${\text{Osc}}_{t,w}\left(f\right)=b_{\delta }\left(t\right)\exp\left(-\frac{{\left(f-f_{\delta }\left(t\right)\right)}^{2}}{2\sigma_{\delta }{\left(t\right)}^{2}}\right)+b_{\theta }\left(t\right)\exp\left(-\frac{{\left(f-f_{\theta }\left(t\right)\right)}^{2}}{2\sigma_{\theta }{\left(t\right)}^{2}}\right).$$

#### 2.7.2 Parameters estimation on a time window

Estimation of the 1/f and oscillatory parameters of the EEG signal S over the time window $W_{w}\left(t\right)$ was done by a fully automated algorithm using the steps described below.

1. The power spectral density of the signal S was computed. (Fig. S5) over $W_{w}\left(t\right)$ using Welch’s method (Welch, 1967) (with a 5 s sub-window). The power spectral density values in the 0.2−15 Hz range were then saved. We used a frequency range up to 15 Hz, to capture the δ and θ activities of interest, and to account for the 1/f decay. Due to the computational cost of the spectral decomposition, we did not use any frequency above 15 Hz. However, since computing the power ratio is not costly, we used a higher upper bound of 20 Hz for computing power ratios (Methods Section 2.6.1).
2. The IRASA method (Wen and Liu, 2016) was used to estimate the 1/f component of the power spectral density in the 0.2−15 Hz range. It consists of applying several scaling factors h on the signal S, computing the corresponding power spectral densities, and the median of the power spectral densities provides the 1/f component. In practice, the signal S in $W_{w}\left(t\right)$ was up-scaled and down-scaled using factors $h_{i}$ between 1.1 and 1.9 with a 0.05 increment and their reciprocals $1/h_{i}$. Then, the geometrical mean ${\text{PSD}}_{GM\left(i\right)}$ of ${\text{PSD}}_{h_{i}}$ and ${\text{PSD}}_{1/h_{i}}$ was computed for each i. Finally, the 1/f estimate was the median ${\text{PSD}}_{m}$ of the ${\text{PSD}}_{GM\left(i\right)}$ for all i (Fig. S5C, light blue curve). The parameters $a_{t},p_{t}$, and $c_{t}$ were obtained by fitting ${\text{PSD}}_{m}$ on the 0.2−15 Hz interval in the log–log scale (Fig. S5C, red curve). The YASA python library (Vallat and Walker, 2021) was used to implement the IRASA method, and the SciPy Python module (Virtanen et al., 2020) to fit parameters $a_{t},p_{t}$, and $c_{t}$.
3. The oscillatory component was computed by removing the estimated 1/f component from the power spectral density (Fig. S5D):
  (13) Osc^t,w(f)=PSDt,w(f)−1/f^t,w(f),
  where 1/f^t,w(f)=atct+fpt.
4. The oscillatory parameters $N_{t}$ and ${\left\{b_{k_{t}},f_{k_{t}},\sigma_{k_{t}}\right\}}_{1\leq k_{t}\leq N_{t}}$ were estimated by fitting a sum of Gaussian functions to the oscillatory component Osc^w,t(f) on the frequency domain 1−15 Hz. This part is a generalization of a method developed in Lindner et al. (2015).
5. Gaussian components where the standard deviation $\sigma_{k_{t}}$ was either smaller than 0.2 or larger than 2 were discarded. In addition, Gaussian components with too small amplitude bkt≤std(Osc^t,w(f)) were discarded. The thresholds 0.2 and 2 were chosen empirically and the threshold std(Osc^t,w(f)) was inspired by Donoghue et al. (2020).
6. At most one Gaussian was selected for which center frequency $f_{k_{t}}$ falls into the θ (resp. δ) 4−10 Hz (resp. 2−4 Hz) band (Fig. S5E). When there were several detected Gaussians that fell into one frequency band, the Gaussian component with the largest area $b_{\theta }=\underset{f_{k_{t}}\in \left[5,10\right]\text{Hz}}{\text{max}}b_{k_{t}}\sigma_{k_{t}}$ (resp. $b_{\delta }=\underset{f_{k_{t}}\in \left[2,4\right]\text{Hz}}{\text{max}}b_{k_{t}}\sigma_{k_{t}}$) was selected.

When no Gaussian component was present in one of the band domains, we considered that there was no prominent rhythm in this band for this time window. In the rare cases where two Gaussians had the same maximum area, we selected the one for which the center frequency $f_{k_{t}}$ was closest to the median frequency, i.e 7.5 Hz for the θ-band, 3 Hz for the δ-band.

#### 2.7.3 Rhythm tracking along GA

Section 2.7.2 estimated the Gaussian $G_{\theta }\left(f,t\right)$ (resp. $G_{\delta }\left(f,t\right)$) of the θ- (resp. δ-) rhythm in one time window of width w=60 s and centered at time t. To define a continuous estimation for the time-varying θ and δ rhythms, the windows $W_{w}\left(t_{i}\right)$ at discretized times $t_{i}$ with a $\frac{w}{2}$ step are used. To define a continuous curve, from these discretized windows $W_{w}\left(t_{i}\right)$, the band Gaussian on $W_{w}\left(t_{i}\right),\;i\in N$ is estimated as described in Section 2.7.2.

When a Gaussian is detected in the consecutive frames $W_{w}\left(t_{i}\right)$ and $W_{w}\left(t_{i+1}\right)$, it is interpolated by a straight line the center frequencies $\left(f_{\text{band}}\left(t_{i}\right),f_{\text{band}}\left(t_{i+1}\right)\right)$ in the time interval $\left[t_{i},t_{i+1}\right]$. The same procedure is also applied to the standard deviation. However, when a band Gaussian is detected in $W_{w}\left(t_{i}\right)$ but none are detected in either $W_{w}\left(t_{i-1}\right)$ or $W_{w}\left(t_{i+1}\right)$, it is not interpolated and the band rhythm is considered not significant.

These steps are applied for both the θ and δ bands. Finally, to further quantify the dynamics of each rhythm, the dynamics of the Gaussian width were followed. The lower and upper curves centered at the frequency f(t) of the fitted Gaussian, each at a standard deviation σ(t) distance were applied so that

$${\text{up}}_{\text{b}}\left(t\right)=f_{\text{b}}\left(t\right)+\sigma_{\text{b}}\left(t\right)$$

(14) $${\text{low}}_{\text{b}}\left(t\right)=f_{\text{b}}\left(t\right)-\sigma_{\text{b}}\left(t\right),$$

where b∈{θ,δ}. Thus the distance between the two curves is precisely twice the variance $\left|{\text{up}}_{\text{b}}\left(t\right)-{\text{low}}_{\text{b}}\left(t\right)\right|=2\sigma_{\text{b}}\left(t\right)$. See Fig. 2G for an example of continuous rhythm tracking, with w=1 min.

#### 2.7.4 Decay of θ center frequency during induction

A common behavior of the θ rhythm was observed in all recordings. At baseline, the θ rhythm is prominent, and its center frequency $f_{\theta }$ is stable around 8 Hz. Then, shortly after the beginning of anesthesia, $f_{\theta }$ decreases rapidly for several minutes (Fig. 2). The start time of the $f_{\theta }$ decay $\tau_{\theta }^{\text{decay}}$ is defined as:

(15) $$\tau_{\theta }^{\text{decay}}=\inf\left\{t>\overset{\text{start}}{\underset{\text{Iso}}{\tau }}\;|\;\text{for all}\;t^{\prime }\in \left[t,t+\text{5 min}\right]\;f_{\theta }\left(t^{\prime }\right)<f_{\theta }\left(t\right)\right\}.$$

#### 2.7.5 Computing the slope of the θ-decay

To estimate the slope of the curve $f_{\theta }\left(t\right)$, we first used the time $\tau_{\theta }^{\text{decay}}$ (Eq. (15)) for which the maximum power of the frequency $f_{\theta }\left(t\right)$ is achieved following anesthesia induction. We used the IRASA decomposition of the signal into the 1/f-decay and oscillatory component. We estimated the slope for each recording by fitting a linear regression to the θ center frequency $f_{\theta }$ on the time interval $\left[\tau_{\theta }^{\text{decay}},\tau_{\theta }^{\text{decay}}+2min\right]$. The slopes were then average per protocol.

### 2.8 Identifying loss and return of movement from EMG

Loss and return of movement were identified by visually inspecting the EMG (Fig. S6). Loss of movement is easily recognizable on the EMG as a switch from active to flat signal. Similarly, the return of movement is a switch from flat to active signal. Notably, the EMG recordings contain artifacts, probably from respiration and electrocardiogram. These artifacts were of very low amplitude and therefore did not impact LOM and ROM identification.

### 2.9 Logistic regression analysis

A logistic regression approach with $l_{2}$ regularization (Bishop and Nasrabadi, 2006) and a regularization constant C =1 were used to evaluate the contribution of several parameters to the prediction of significant time spent in IES. The criterion for class separation was chosen with the γ rebound phenomena, which happens for recordings with more than 30 s in IES (Section 3.3). The dataset was thus divided into a positive class (total time spent in IES is more than 30 s) and a negative class (total time spent in IES is less than 30 s).

The dataset was separated into training and validation sets using a stratified group k-fold strategy: this strategy ensures that recordings from one individual are not split between the train and validation set (group) and that the validation sets of all folds have equivalent proportions of positive and negative labels (stratified). The scikit-learn python library (Pedregosa et al., 2011) was used with a 4 folds separation, due to the limited number of recordings in the negative class.

The logistic regression models were fed timestamps of events identified in Section 3.4. Due to constraints on the various durations, each time feature was normalized given to the model in the following way:

(16) $$\tau^{*}=\text{log}\left(1+\frac{100}{\tau -\tau_{\text{Iso}}^{\text{start}}+0.1}\right),$$

where τ is the time feature computed by our algorithm, τ∗ is the feature fed to the logistic regression model, and $\tau_{\text{Iso}}^{\text{start}}$ is the time of the beginning of anesthesia. This choice was inspired by Sun and Holcman (2022).

### 2.10 γ-rebound identification

The γ-rebound recordings were identified when the γ-power was significantly larger during recovery than in the rest of the recording for at least 2 min, using a threshold $T_{\gamma }$ on the power defined by

$$T_{\gamma }=3\;{\text{RMS}}_{\text{BL}},$$

$${\text{RMS}}_{\text{BL}}=\text{RMS}\left({\left(p_{\gamma }\left(t\right)\right)}_{0\leq t\leq \tau_{\text{Iso}}^{\text{start}}}\right),$$

where $p_{\gamma }\left(t\right)$ is the power of the filtered 50−70 Hz EEG signal, computed on sliding windows of width 0.2 s and zero overlap and $\tau_{\text{Iso}}^{\text{start}}$ is the instant where the isoflurane starts to be administered.

**Fig. 3:**
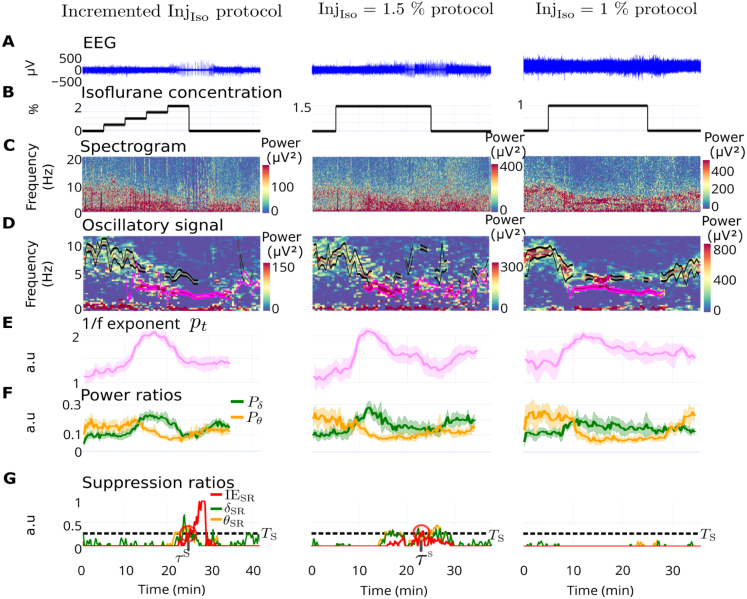
**Influence of anesthesia protocols on spectral dynamics. (A)** Representative EEG examples of three GA protocols **(B)**. **(C)** Spectrograms.**(D)** Oscillatory components and automatically tracked θ- (gray and black) and δ- (pink) rhythms. **(E)** Average exponent $p_{t}$ of the 1/f-component $\frac{a_{t}}{c_{t}+f^{p_{t}}}$ per protocol. **(F)** Average power ratios $P_{\delta }$ (green) and $P_{\theta }$ (yellow) per protocol. **(G)** Examples of suppression ratios computed for the δ-band ($\delta_{\text{SR}}$), θ-band ($\theta_{\text{SR}}$) and the IES (${\text{IES}}_{\text{SR}}$). Error bands indicate the 95% confidence intervals computed using the t-distribution.

## 3 Results

### 3.1 Higher isoflurane concentration increases IES incidence

To evaluate whether the GA protocol could impact the appearance and distribution of IES, we implemented three different protocols with varying isoflurane concentrations. In the first, which we refer to as the incremented protocol, the isoflurane concentration was gradually increased from 0.5% to 2% every 5 min in 0.5% increments (n = 13). In the other protocols the isoflurane concentration was fixed at 1% (n = 9), and 1.5% (n = 8) (Fig. 1A–B). We refer to these protocols as the 1% protocol and the 1.5% protocol respectively.

To detect IES and suppressions of the prominent bands the θ-band (4−10 Hz) and the δ-band (0−4 Hz), we used an automated algorithm adapted from Cartailler et al. (2019) (Fig. 1C and Methods). We computed the suppression ratios ${\text{IES}}_{\text{SR}}$, $\theta_{\text{SR}}$ and $\delta_{\text{SR}}$, which we defined as the duration ratio spent in iso-electric suppression, θ-suppression, and δ-suppression respectively, over a 20-second sliding time window (Fig. 1D and Methods). We then identified the delay $\tau_{\theta \text{S}}^{1}$ to the first θ-suppression occurrence, the delay $\tau_{\text{IES}}^{1}$ to the first IES occurrence, and the delay to start of long IES $\tau^{S}$ (defined as the time at which the iso-electric suppression ratio ${\text{IES}}_{\text{SR}}$ exceeds the empirically chosen threshold $T_{S}=$ 0.25) (Fig. 1E and Methods). Using the same sliding-window analysis, we also computed the time-dependent relative powers of the δ- and θ- bands (see Methods).

For the incremented protocol, IES appeared uniformly in 100% (13/13) of mice, 16.5 ± 2.2 min after GA onset, at 2% isoflurane concentration. When using 1.5% concentration in a subset of the same mice, IES appeared in 89% (8/9) of mice, 10.7 ± 1.8 min after GA onset. However, at 1% isoflurane concentration, our algorithm detected IES in only 62.5% (5/8) of mice, 12 ± 4 min after GA onset (Fig. 1F). We found a statistical difference in the cumulative IES duration $\Delta_{\text{IES}}$ (defined as the cumulative time spent in IES per recording) between the incremental protocol and the 1% protocol but not between other protocols (Fig. 1G). Interestingly, the θ-band was suppressed at the same time as IES, but not before (Fig. 1C2, Fig. 1H), contrasting with propofol-induced GA in humans, where frontal α-suppressions precede IES (Cartailler et al., 2019, Sun and Holcman, 2022). At this stage, we conclude that suppressions of the θ- and δ-bands do not reliably precede IES appearance in primary somatosensory EEG recordings from mice during isoflurane-induced GA.

### 3.2 Spectral decomposition reveals θ- and δ- bands predominance

In analyzing the EEG recordings, we adopted a spectral analysis approach using the IRASA algorithm (see Methods). This algorithm provides a robust estimation of the 1/f-component present in the EEG signal, particularly when additional oscillatory components are present in the low-frequency domain. Using this approach, we demonstrated the continuous estimation of parameters for the 1/f- and oscillatory components in the EEG recordings, a task not easily achieved with wavelet decompositions alone.

The decomposition began with a single time window $W_{t}$ (Fig. 2A). For each window $W_{t}$, the power spectral density was separated into two components. The first component was the 1/f-component, representing a decaying trend fitted by the function $y_{1}\left(f\right)=\frac{a_{t}}{c_{t}+f^{p_{t}}}$ (Fig. 2A2, see Methods). The second component was the oscillatory component (Fig. 2A3), accounting for activity in isolated frequency bands, fitted with Gaussians (Fig. 2A4). This separation was extended across the entire recording by sliding the time window $W_{t}$, (Fig. 2B–D). The algorithm successfully separated the oscillatory component and continuously tracked it over time (Fig. 3D, see Methods). In isoflurane-induced GA in mice, two active frequency bands were identified: the θ- and δ-rhythms (illustrated in Fig. 3D as black and pink lines, respectively). The θ-rhythm was consistently present before GA induction and decayed shortly after induction, while the δ-rhythm appeared a few minutes after GA induction. The dynamics of the 1/f-exponent $p_{t}$ revealed a general trend across all recordings and protocols: $p_{t}$ increased during the beginning of GA, reached a plateau, and then decreased (Fig. 3E and supplementary section S1.2).

These band dynamics were also observable in the raw signal without spectral decomposition. Notably, the relative power of the θ-band $P_{\theta }$ (yellow) was consistently higher than the relative δ power $P_{\delta }$ (green) before the beginning of GA (Fig. 3F). Shortly after GA induction, the power ratio $P_{\theta }$ decayed, while $P_{\delta }$ increased, leading to a reliable change in band dominance, a feature used for classifying distinct GA states. Subsequently, $P_{\delta }$ decreased while $P_{\theta }$ remained low until the end of GA. The power $P_{\delta }$ showed a weak correlation with the coefficient $p_{t}$ (Fig. S7). Finally, the iso-electric suppression ratio tended to be higher during the incremented protocol compared to the others (Fig. 3G). In conclusion, the θ- and δ-bands exhibit prominence during GA, displaying comparable behaviors across protocols.

### 3.3 Higher levels of IES precede the appearance of a γ pattern during recovery from GA

**Fig. 4:**
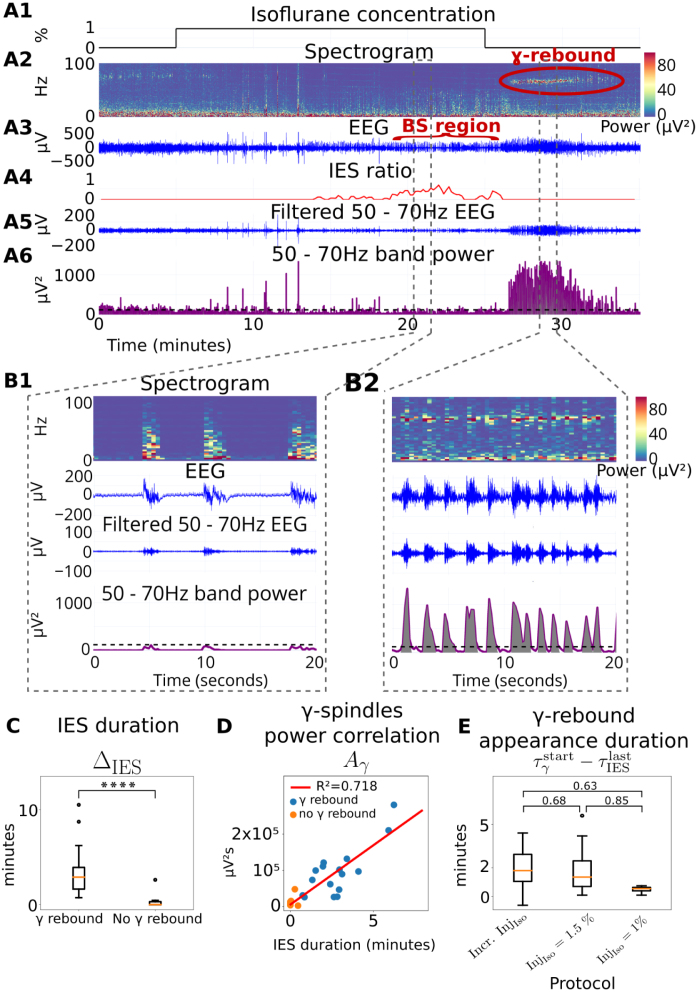
**Cumulative IES duration**>**30 s precedes**γ-**rebound during recovery from GA. (A)** Spectral analysis of γ-rebound (red circle) that appear during recovery after burst suppression (BS region, red) characterized by IES ratio increase. γ-rebound shows significant amplitude in the 50−70 Hz power band, above threshold $T_{\gamma }$ (black dashed lines). Magnification of 20-s plots **(B1)** during burst suppression and **(B2)** during γ-rebound. **(C)** Statistics of IES duration in separated groups with and without gamma rebound, ****p<0.0001 (two-sided Mann–Whitney U test). **(D)** Boxplot of γ-rebound appearance duration after the last IES given for the three anesthetic protocols and p-values of the associated two-sided Mann–Whitney U test. **(E)** Correlation between areas under γ-bursts power (gray area in B2) and IES durations.

In some recordings, the EEG spectrogram during GA recovery revealed a stable and long-lasting activity in the γ-frequency domain (50−70 Hz range) (Fig. 4A). This phenomenon which we refer to as γ-rebound, is characterized by a γ-power greater than that before and during GA (Methods Section 2.10, Fig. 4A2,6).

To identify spectral features that would predict γ-rebound occurrence, we focused on IES. We noticed that γ-rebounds appeared after GA with burst suppression episodes (Fig. 4A3–4). Burst suppressions were identified visually as an alternation of IES and burst activity in the 0−50 Hz range (Shanker et al., 2021) (Fig. 4B1). In parallel, the γ-rebound consisted of a succession of high amplitude and high power (purple) bursts located in a narrow frequency range around 60 Hz (Fig. 4B2).

To further characterize γ-rebound, we investigated whether it was associated with more time spent in IES. We found that γ-rebound was present in recordings where the cumulative IES duration ($\Delta_{\text{IES}}$) was greater than 30 s (Fig. 4C). Thus, recordings were differentiated into two groups (Methods), those with and without γ-rebound, for which the cumulative IES duration was 198 ± 144 s and 6.6 ± 10.2 s respectively.

Finally, we investigated the relationship between the cumulative IES duration ($\Delta_{\text{IES}}$) and the power of the γ-rebound. To do so, we computed the instantaneous power $p_{\gamma }$ in the 50−70 Hz range (Fig. 4A6). We then computed the area $A_{\gamma }$ under the curve of $p_{\gamma }$ from the end of GA at time $\tau_{\text{Iso}}^{\text{stop}}$ to the end of the recording (time $\tau_{\text{rec}}^{\text{stop}}$). The distribution of duration and area $\Delta_{\text{IES}}$, $A_{\gamma }$ (Fig. 4D) was fitted with a linear regression y=ax+b, where we found a=32757 μV${}^{2}$, b=6047 μV${}^{2}$s, and $R^{2}=0.72$. This shows that the γ-burst power during recovery was highly correlated with the cumulative time spent in IES and thus could be used to mirror the depth of anesthesia. Additionally, we found no correlation between $\Delta_{\text{IES}}$ and the γ-rebound duration, or the γ-power $p_{\gamma }$ (SI section S1.4 and Fig. S8).

We then studied the time of appearance of the γ-rebound relative to the last episode of IES. We found that the γ-rebound appeared a few minutes after the last IES (Fig. 4E), with no statistical differences across protocols. To conclude, we propose that γ-rebound is an *a posteriori* marker of long IES, which is characteristic of too deep anesthesia (see Table 2).

**Table 2:** Timestamps of EEG and EMG time-frequency events.

Timestamp	Symbols
Starting time of recording	$\tau_{\text{start}}^{\text{rec}}$
Start of isoflurane administration	$\tau_{\text{Iso}}^{\text{start}}$
Stop isoflurane administration	$\tau_{\text{Iso}}^{\text{stop}}$
Stop recording session	$\tau_{\text{rec}}^{\text{stop}}$
Time of decay of θ center frequency $f_{\theta }$	$\tau_{\theta }^{\text{decay}}$
Appearance time of δ rhythm	$\tau_{\delta }^{\text{app}}$
Dampening time of θ rhythm	$\tau_{\theta }^{\text{disp}}$
Dampening time of δ rhythm	$\tau_{\delta }^{\text{disp}}$
First IES appearance time	$\tau_{\text{IES}}^{1}$
First significant IES time	$\tau^{\text{S}}$
γ rebound begins	$\tau^{\gamma }$
Loss of movement time	$\tau^{\text{LOM}}$
Return of movement time	$\tau^{\text{ROM}}$

**Fig. 5:**
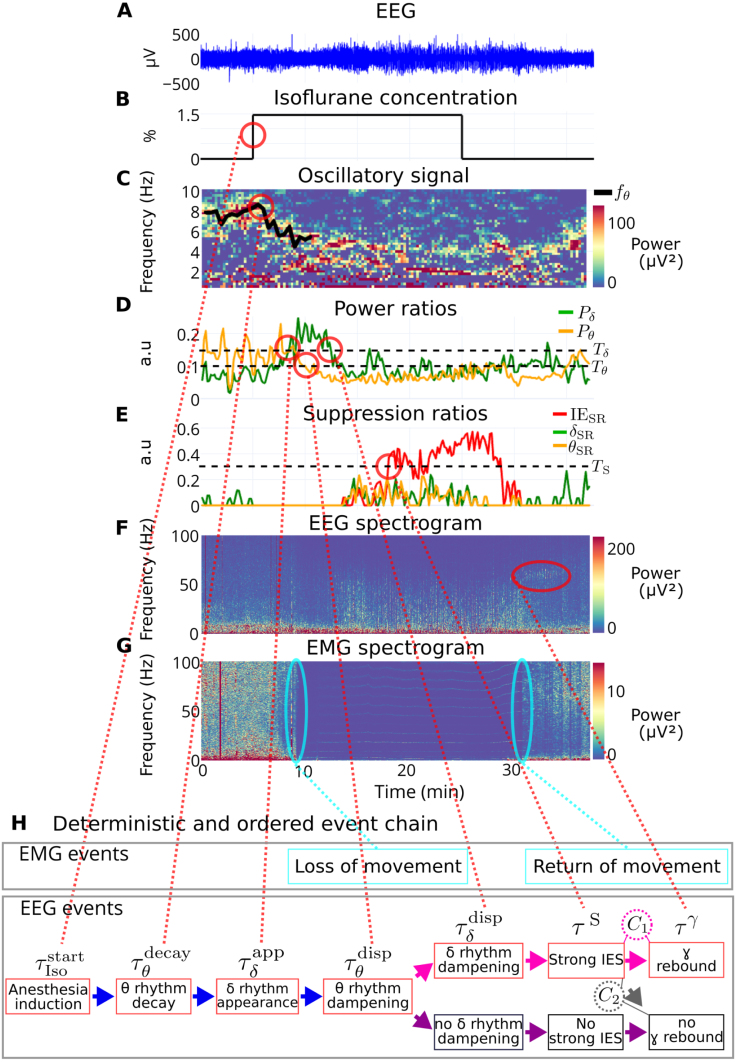
**Ordered and chain of time-frequency events. (A)** EEG recording. **(B)** Anesthesia protocol. **(C)** Extracted oscillatory signal and tracking of the center frequency $f_{\theta }$ of the θ rhythm. **(D)** Band power ratios. **(E)** Suppression ratios. **(F)** EEG spectrogram. **(G)** EMG spectrogram. **(H)** Deterministic and ordered frequency events chain. $C_{1}$: cumulative IES time > 30 s. $C_{2}$: cumulative IES time < 30 s.

### 3.4 Transient sequence of EEG time-frequency patterns during isoflurane-induced GA

In this section, we incorporate the spectral decomposition (Section 3.2) and the γ-rebound (Section 3.3) to the EEG segmentation. To study whether EEG data (Fig. 5A–B) could be segmented based on band tracking (Fig. 5C), power ratios (Fig. 5D), band suppressions (Fig. 5E), IES, EEG and EMG spectrograms (Fig. 5F–G), we identified several key events which reliably appear in a systematic temporal order.

The first relevant event was the beginning of GA, characterized by isoflurane delivery in the air (Fig. 5H, first red box) at time $\tau^{\text{start}}\text{Iso}$. We found a decay of the θ-band, characterized by a decrease of the θ center frequency fθ (Fig. 5C). We refer to the beginning time of this phase as $\tau^{\text{decay}}\theta$ (Eq. (15), Methods). To quantify this decay, we used the IRASA decomposition to follow the θ−oscillatory component. We then computed the slope of the θ-component in the time-frequency domain from the maximum of the θ−frequency after induction which was persistently decaying over few minutes. A regression over 2 min lead to the following negative slopes S computed for each protocol: $S_{\theta 1\text{\%}}-1.04\pm 0.465$ (n = 9), $S_{\theta 1.5\text{\%}}=-1.02\pm 0.543$, (n = 8) and $S_{\theta \text{step}}=-0.746+/-0.467$ (n = 13) in Hz/min, thus confirming this general decay trends.

Third, we found that the δ-rhythm appeared at time $\tau_{\delta }^{\text{app}}$ (Eq. (8), Fig. 5D). Fourth and fifth, the θ-rhythm dampened at time $\tau_{\theta }^{\text{disp}}$, while the δ- rhythm also dampens at time $\tau_{\delta }^{\text{disp}}$ (Eq. (9), Fig. 5D). Significant IES emerged at time $\tau^{\text{S}}$ (Eq. (6), Fig. 5E). Finally, we observed a γ-rebound at time $\tau^{\gamma }$ (Method Section 2.10, Fig. 5G).

Although we identified a protocol-independent, ordered chain of EEG events during induction and maintenance of anesthesia, we were looking for a possible signature during the emergence phase of GA. However, we found that the θ− and δ− rhythms did not have a specific characteristic and systematic trends during emergence as shown in Fig. S7A–B. Indeed, plotting the θ− and δ− relative powers reveals opposite trends: for the incremented and 1.5% protocols (left and center columns, Fig. S7A), the relative delta power (green plot) increases to a value higher than the baseline value, while the relative theta power increases very softly (yellow plot). Similar conclusion can be reached from plotting the ratio $R_{\theta /\delta }=\frac{P_{\theta }}{P_{\delta }}$ or the time-averages values ${\overset{¯}{R}}_{\theta /\delta }=\langle \frac{P_{\theta }}{P_{\delta }}\rangle$ over the emergence phase, leading to ${\overset{¯}{R}}_{\theta /\delta }=1.067$ for the step protocol, 1.056 for the constant 1.5% protocol, and 0.851 for the constant 1% protocol. At this stage, we conclude that the δ- and θ- rhythms do not have a general trends during the emergence phase, as shown by the relative powers, power ratios over time or the average computed over this period (SI section S1.1).

In summary, we identified here EEG events (Fig. 5H) that are related to brain anesthesia depth. These events seems to occur in the same temporal order across all recordings, represented by the time inequalities (see Table 2 for definition)

(17) $$\tau_{\text{Iso}}^{\text{start}}<\tau_{\theta }^{\text{decay}}<\tau_{\delta }^{\text{app}}<\tau_{\theta }^{\text{disp}}<\tau_{\delta }^{\text{disp}}<\tau^{\text{S}}<\tau^{\gamma }.$$

Finally, we quantified the duration between these EEG events and found that the time order defined by Eq. (17) was valid in all recordings, although the duration between two consecutive events varied across protocols (Table 1). We concluded that, to reach a specific event, the anesthetized brain passed through all preceding states in a systematic order. For instance, significant IES only occurred for recordings in which the δ band had dampened (SI section 1.6 and Fig. S9).

We further decided to investigate whether there was any correlation between these EEG events and mice movement. Using the EMG, we identified the time $\tau^{\text{LOM}}$ of movement loss and the return of movement time $\tau^{\text{ROM}}$ (Methods Section 2.8, Fig. S6, and Fig. 5G). We found that the θ-rhythm dampening and the LOM time were very close, such that this difference was $\tau_{\theta }^{\text{disp}}-\tau^{\text{LOM}}=1.9\pm 4.1$ min. Likewise, when a γ-rebound appeared in the EEG, it started with ROM, characterized by a small difference $\tau^{\gamma }-\tau^{\text{ROM}}=0.3\pm 0.8$ min. Interestingly, the γ-rebound was not due to EMG contamination (SI section S1.5 and Fig. S10). To conclude, we reported here a sequence of key, strictly ordered events occurring during GA, which was protocol-independent, with some variability in the transition durations between two consecutive events. This chain of events was characterized by the dynamics of the δ- and θ-bands. This sequence of events revealed two possible EEG behaviors: one leading to long IES and γ-rebound, and another characterized by little or no IES and the absence of γ-rebound.

### 3.5 Predictive analysis and state-chart decomposition of isoflurane-induced GA

To further quantify the predictive value of the transient timestamps identified in Section 3.4, we used a logistic regression (Methods Section 2.9) to determine whether the delay to first IES occurrence time $\tau_{\text{IES}}^{1}$, the delay to θ-band decay time $\tau_{\theta }^{\text{decay}}$, the delay to the appearance $\tau_{\delta }^{\text{app}}$ of the δ-band, the delay to dampening of the θ-band (time $\tau_{\theta }^{\text{dis}}$), and the delay to dampening the δ-band at time $\tau_{\delta }^{\text{dis}}$ enable a reliable prediction of IES. More specifically, we defined IES sensitivity as whether $\Delta_{\text{IES}}$ exceeds 30 s during GA, based on the γ-rebound analysis (Section 3.3).

**Fig. 6:**
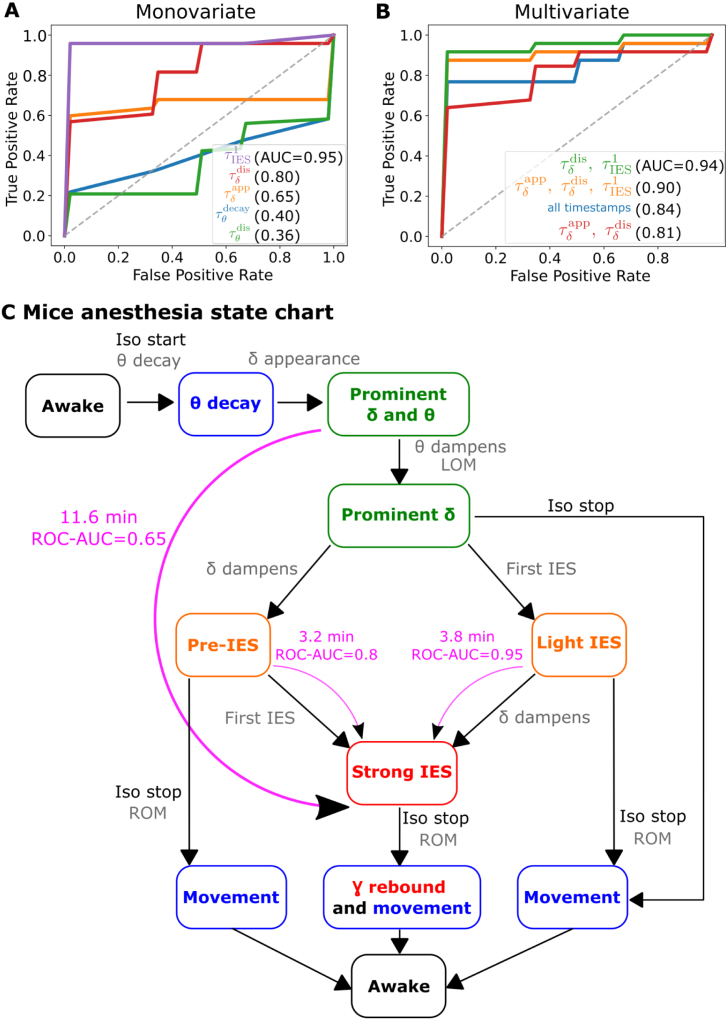
**Logistic regression and state chart representation.** IES Sensitive vs non-IES sensitive classification based on θ, δ and IES parameters. ROC curves and AUC are computed using a logistic regression classifier for **(A)** single predictors and **(B)** various combinations of predictors. **(C)** State chart characterizing the transitions between the different GA states starting from the awake state (black). Further states are associated with motion (blue), moderate depth of anesthesia (green), intermediate depth of anesthesia (orange) and high depth of anesthesia (red). They are characterized by the presence/absence of the δ and θ rhythms and IES. There are three main predictive states of IES sensitivity: the state “Prominent δ and θ” with a predictive power ROC-AUC = 0.65 and an average time delay to strong IES of 11.6 min, the state “Pre-IES” (ROC-AUC = 0.8 and average time of 3.2 min), and finally the state “Light IES” with a predictive power ROC-AUC = 0.95, and an average time of 3.8 min.

The univariate classification (Fig. 6A) logically revealed that the variable $\tau_{\text{IES}}^{1}$ was most predictive of IES sensitivity, with a receiver operating characteristic area under the curve (ROC-AUC) value of 0.95. Interestingly, the time $\tau_{\delta }^{\text{dis}}$ ($\tau_{\delta }^{\text{app}}$ respectively) also carried a predictive power, characterized by a ROC-AUC value of 0.8 (0.65 respectively). The duration $\tau^{S}-\tau_{\delta }^{\text{dis}}$ was 1.9 ± 1.8 min for the incremented isoflurane protocol, and 4.5 ± 3.1 min for the 1.5% protocol (SI section 1.6). Furthermore, the regression analysis showed that the times $\tau_{\theta }^{\text{decay}}$ and $\tau_{\theta }^{\text{dis}}$ carried much less predictive power with ROC-AUC values of 0.4 and 0.36 respectively. We thus conclude that the δ-band dynamics carry more predictive value than the θ-band with respect to IES sensitivity.

We then applied a multivariate logistic regression (Fig. 6B) to evaluate the predictive power of specific timestamps combinations. We found that the three models that performed well were trained the couple ($\tau_{\text{IES}}^{1},\tau_{\delta }^{\text{dis}}$) (ROC-AUC = 0.94), on the triplet ($\tau_{\text{IES}}^{1},\tau_{\delta }^{\text{dis}},\tau_{\delta }^{\text{app}}$) (ROC-AUC = 0.9), or on all the timestamps (ROC-AUC = 0.84). Finally, with the model trained only on δ-band appearance and dampening, we observed lower performance with ROC-AUC = 0.81. From these analysis, we conclude that the most predictive variables are the first IES time $\tau_{\text{IES}}^{1}$, the δ-appearance time $\tau_{\delta }^{\text{app}}$, and the δ-disappearance time $\tau_{\delta }^{\text{dis}}$. We also note that none of the multivariate models outperformed the univariate model trained on the first IES time $\tau_{\text{IES}}^{1}$ (ROC-AUC = 0.95).

To account for these deterministic relationships (Fig. 5H and Table 1), we organize these results into a state chart diagram as a synthetic graphical representation of the different states we previously reported: each state is characterized by θ- and δ- band spectral properties and suppression ratios (Fig. 6C). The initial state of the state chart, called “Awake”, is defined by a prominent θ-band with a stable θ center frequency $f_{\theta }$ (Methods Eq. (12)), no prominent δ-band, and no IES. Subsequently, when isoflurane inhalation starts, the EEG switches to the second state called “θ decay”, in which $f_{\theta }$ decreases. The third state is “Prominent δ and θ”, where the δ-rhythm has appeared (Methods Eq. (15)). The fourth state is “Prominent δ”, where θ has dampened. Three states are accessible from there, which we detail below. In “Pre-IES”, the θ- and δ- bands are dampened and there is no IES. In “Light IES”, the θ-band is inactive, the δ-band is active, and there is little IES ($0\leq {\text{IES}}_{\text{SR}}\leq 0.25$). The third state accessible from “Prominent δ” is called “Movement”, following ROM during recovery after isoflurane cessation. The next state after “Pre-IES” and “Light IES” is “Strong IES”, characterized by high ${\text{IES}}_{\text{SR}}$ values (Methods Eq. (6)), and should be avoided. When GA stops, ROM happens, leading to the “γ-rebound and movement” state. If isoflurane stops in a different state than “Strong IES”, the EEG transitions to the “Movement” state without γ-rebound.

Interestingly, our statistics revealed an 11.6 min delay (in average) between the states “Predominant δ” and “Strong IES”. This transition has a predictive value (ROC-AUC = 0.65). Moreover, the average transition time from the δ-rhythm disappearance state to the strong IES state is reduced to 3.2 min with a higher predictive (ROC-AUC = 0.8). Finally, the transition from the light IES state to the strong IES state occurs in 3.8 min on average, with a very high predictive value (ROC-AUC = 0.95).

In summary, we constructed a state chart associated with the depth of anesthesia, where each state is characterized by parameters computed from the EEG and EMG signals. Interestingly, the regression analysis revealed that the first IES time $\tau_{\text{IES}}^{1}$, the δ-appearance time $\tau_{\delta }^{\text{app}}$, and the δ-dampening time $\tau_{\delta }^{\text{dis}}$ could be used to predict IES sensitivity and thus provide three different checkpoints that could be used in real-time analysis to monitor the brain IES sensitivity. These states were interlaced with clinical events observed during GA, namely GA induction, GA cessation, and loss and return of movement.

## 4 Discussion

Improving the monitoring of the depth of anesthesia was a crucial endeavor aimed at reducing post-operative complications. While addressing anesthetic overdose is essential, preventing overdose has proven elusive with current clinical EEG-based monitors. In our study, we employed a signal processing approach combined with classification techniques, offering the potential to enhance the predictability and prevention of overdose. Through this research, we formulated a computational method grounded in EEG signals, and established an automated and interpretable pipeline. Our approach uncovered reproducible and timely ordered brain states and transitions, providing a comprehensive characterization of the entire process of general anesthesia, spanning from induction to emergence. To distill this wealth of information, we synthesized these states and transitions into a state-chart representation that reflects the real-time depth of anesthesia and predicts the onset of iso-electric suppression (IES) several minutes in advance. This predictive capability holds the promise of preventing overdose, a task that remains unattained by the existing clinical EEG-based monitors.

In our study, we performed a time-frequency spectral analysis on EEG recordings obtained during isoflurane-induced general anesthesia (GA) in mice. This analysis involved decomposing the power spectrum density to identify prominent band activities. Notably, these activities were found in the θ- and δ-domains during general anesthesia and in the θ-, δ-, and γ-domains during the recovery phase. To further understand the dynamics, we calculated the relative power of the θ and δ bands. The separation of oscillatory components from the 1/f-decay was achieved using the IRASA algorithm (Wen and Liu, 2016). Simultaneously tracking several frequency rhythms, we extracted their associated Gaussian parameters (refer to Fig. 2 for visualization). Finally, we employed a threshold heuristic to detect iso-electric suppressions (IES) and quantified the cumulative time spent in this state.

Employing this method enabled the identification of a reliable sequence of events (Fig. 5), delineating the entire process of isoflurane anesthesia, spanning from induction to emergence. As a result, we formulated an EEG state chart that provides a comprehensive reflection of the duration and anesthesia levels (Fig. 6C), despite the underlying physiological mechanism remaining unknown. This state chart holds the potential for precise anesthetic titration, allowing for the avoidance of undesirable states associated with prolonged iso-electric suppressions (IES). Notably, we discovered that the dynamics of the δ-band served as a predictive indicator for long IES several minutes in advance. In contrast, despite the activity of the θ-band during isoflurane general anesthesia (GA), it exhibited no predictive value concerning long IES. Our hypothesis suggests that θ-band activity may correlate with animal movement, given its dampening around the same time as the loss of movement (LOM).

Additionally, our observations indicated that the trajectory of EEG during recovery did not mirror the sequential events observed during induction and maintenance of GA, highlighting an asymmetry in the recovery process. Interestingly, we identified a specific activity in the γ-band during recovery, termed γ-rebound. Notably, this pattern manifested in recordings where cumulative IES time exceeded 30 s. Consequently, we propose that this γ-rebound pattern could serve as a retrospective marker of deep anesthesia.

### 4.1 Beyond the power spectral EEG decomposition

During general anesthesia, the EEG signal combines band oscillations with a prevalent 1/f-decay. Various brain regions contribute to the oscillatory bands, while the 1/f-decay typically represents the overall spontaneous neuronal firing activity (Buzsáki et al., 2012-06, Buzsáki and Mizuseki, 2014, Buzsáki, 2006). Decomposing the power spectral density of neurophysiological signals offers a means to extract parameters and quantify these distinct signals (Ouyang et al., 2020). This process involves estimating the 1/f- and oscillatory components directly on the power spectral density and fitting Gaussians to the oscillatory components, a procedure facilitated by the FOOOF algorithm (Donoghue et al., 2020). Extracted features, such as the maximum amplitude frequency and band power, can then be utilized to quantify the prominence of α-oscillations and the 1/f-exponent, revealing differences between young and elderly patients (Donoghue et al., 2020). However, this approach comes with several limitations. Notably, when applied to study the loss of consciousness (LOC) in humans anesthetized with propofol, it failed to detect oscillatory activity in the δ-domain (Brake et al., 2021). To address this misclassification, a convolution procedure on the EEG neuronal network was proposed (Brake et al., 2021). In our work, we sought to overcome the FOOOF algorithm’s low performance in the low-frequency domain. We achieved this by estimating the 1/f-component using irregular sampling auto-spectral analysis (Wen and Liu, 2016), employing successive re-samplings of the EEG signal directly, rather than in the frequency domain. Subsequently, we parameterized the remaining oscillatory component with Gaussians (Lindner et al., 2015). Importantly, our estimation method dynamically tracked the time evolution of the δ- and θ-bands simultaneously (Fig. 2G). The algorithm developed in this study enables a robust and dynamic spectral decomposition, revealing the band characteristics during general anesthesia. This fully automated algorithm stands as a versatile tool easily applicable to other datasets.

### 4.2 Automated threshold selection for IES detection

In coma and general anesthesia (GA), prolonged iso-electric suppression (IES) episodes have been linked to subsequent complications such as confusion, delirium, or memory loss (Andresen et al., 2014, Fritz et al., 2016b). Recognizing the need for robust and automated real-time IES detection, various methods have been employed. Typically, the identification of long IES involves computing the fraction of time spent in IES within a sliding time window, often referred to as the burst suppression ratio (Rampil et al., 1988), akin to our present IES ratio ${\text{IES}}_{\text{SR}}$. Similarly, the burst suppression probability (BSP) (Chemali et al., 2013) provides binary segmentation indicating the presence of IES. However, many existing algorithms (Rampil et al., 1988, Chemali et al., 2013, Cartailler et al., 2019) rely on a fixed threshold value that does not account for inter-individual variability in EEG amplitude (Shanker et al., 2021).

To address this limitation, some approaches, such as estimating the variance of the EEG signal iteratively (Westover et al., 2013), attempt to correct the threshold based on the signal’s variance. However, this method requires expert adjustment of model parameters for each patient, limiting its practicality. Another direct approach (Sun and Holcman, 2022) involves computing the minimum between a fixed threshold value and the median of the difference between the upper and lower envelope of the signal, considering individual signal variability. Nevertheless, this approach still incorporates a fixed threshold value.

Our data-driven approach represents a significant advancement in automatic and accurate IES detection. We propose to compute the IES threshold as $T_{\text{IES}}=r_{\text{IES}}\text{RMSEEG}$, with the coefficient rIES accommodating inter-individual variability. The selection of the relative threshold $r_{\text{IES}}$ was meticulously performed by continuously exploring detected IES duration concerning $r_{\text{IES}}$ (SI sec.2.1 and Fig. S2). This approach has demonstrated robustness on the current dataset, and further evaluation of the optimal relative threshold value $r_{\text{IES}}=0.7$ on a larger dataset would be valuable. Importantly, this heuristic could be adapted for real-time applications by assessing the signal RMS(t) before the onset of GA as opposed to during GA.

### 4.3 Revisiting the landmarks to control GA

The current approach broadens the scope of monitoring the depth of anesthesia beyond iso-electric suppressions (IES). Anticipating the occurrence of IES in advance holds the potential to reduce the incidence of post-anesthesia complications associated with IES. However, this task remains challenging due to the absence of a physiological model capable of predicting IES based on the EEG signal.

Recent studies (Cartailler et al., 2019, Sun and Holcman, 2022) have highlighted three parameters predictive of IES sensitivity in frontal EEG recordings of the human brain under propofol. These parameters include the first appearance time of an α suppression, the slope of the α-suppression ratio, and the delay to the first IES occurrence. In contrast, our observations in primary somatosensory EEG recordings during isoflurane-induced general anesthesia (GA) in mice revealed that band suppressions were not predictive of IES sensitivity, as they did not reliably precede IES. Instead, we identified three parameters predictive of IES sensitivity: the appearance time of the δ-band, the dampening time of the δ-band, and the time to the first occurrence of IES. Notably, the δ relative power appeared to be predictive, analogous to α suppressions observed in humans under propofol. This discrepancy in predictive parameters may arise from differences in the drugs used (Kenny et al., 2014), the species recorded (human vs. rodent), or the electrode locations employed.

### 4.4 Roles of δ- and θ- oscillations in isoflurane-induced GA

In the context of isoflurane-induced general anesthesia (GA) in mice, our findings reveal oscillatory activity in the δ- and θ-frequency domains. These two bands appear to serve distinct functions: the dynamics of the δ band are linked to neuronal responses to isoflurane and can anticipate iso-electric suppressions (IES), while the θ dynamics seems associated with mouse movements, showing no statistical correlation with IES. Intriguingly, after GA onset, the δ rhythm manifests suddenly, whereas the θ rhythm exhibits a gradual decay.

In humans anesthetized with propofol, the immediate appearance of δ rhythm synchronizes with the loss of consciousness (Brake et al., 2021). Similarly, high doses of sevoflurane induce coherent δ oscillations in rats (Guidera et al., 2017). Our results further indicate that δ oscillations are present during deep anesthesia, and under fixed protocols, the timing of their appearance and disappearance proves predictive of prolonged IES.

While θ oscillations in the rodent hippocampus are typically associated with exploratory locomotion (Vanderwolf, 1969, Welsh et al., 1985), the θ oscillations observed in the cortex in our study likely do not originate from the hippocampus. Recent research has identified a neural rhythm known as the respiration-entrained rhythm (Tort et al., 2018), observable across various brain regions, peaking at the same frequency as breathing (around 12 Hz during exploration and 3 Hz during REM sleep). It is plausible that some of the oscillatory activity observed here could stem from the respiration-entrained rhythm.

In conclusion, the state chart diagram (Fig. 6C) encapsulates the intricate possible pathways of the brain during isoflurane-induced GA, which could potentially be generalized to other anesthetics.

### 4.5 Characteristic γ−activity during recovery from GA

A rebound activity in the γ-band appeared and persisted for several minutes after GA cessation (Fig. 4). This specific pattern only happened in recordings with cumulative IES duration exceeding 30 s. Increased γ activity has been documented during awakening from 2% isoflurane GA in rats, but no γ-rebound pattern was reported in Kortelainen et al. (2012). The isoflurane protocol used during recovery in Kortelainen et al. (2012) differs from ours, which could explain the absence of γ-rebound. We showed here that this γ-rebound was highly correlated with the presence of long IES (Fig. 4E). We therefore hypothesize that the γ-rebound could reflect a neuronal network rebound, after a long period of hyperpolarization. The exact physiological mechanism underlying this manifestation remains to be clarified.

### 4.6 Potential clinical implications

The real-time monitoring of depth of anesthesia in human patients relies on EEG recording, with monitors processing the EEG and displaying an index between 0 and 100. Lower index values indicate deeper sedation, while higher indices suggest lighter sedation or wakefulness. Extensive observational studies have established robust statistical associations between low EEG index values and poor outcomes (Sessler et al., 2012). However, the implementation of simple alerts for undesired EEG states has not shown an improvement in outcomes, potentially due to the lack of pharmacokinetic profiles for current hypnotic drugs (Sessler et al., 2019). Correcting an overdose once detected by the EEG monitor requires a delay of several minutes, emphasizing the need for preventive measures against undesired EEG states. Therefore, new methods for analyzing and interpreting EEG states in routine clinical practice are deemed necessary.

In our research, we identified robust transition states between desired and undesired EEG states and developed analytical tools for automatic detection of these transition states in mice. Notably, we successfully identified transition states preceding iso-electric suppressions (IES), a typical undesired EEG state associated with poor outcomes (Pawar and Barreto Chang, 2022, Shanker et al., 2021). This prediction contrasts with current EEG monitors, where IES episodes may occur even with index values within the desired range (Bruhn et al., 2000, Besch et al., 2011). In summary, the real-time identification of transitions holds the potential to prevent these undesired states. Further studies are essential to validate the robustness of these transition states in both mice and humans undergoing GA. Additionally, this approach warrants further validation using other GA protocols in which EEG and EMG states are parallel with behavioral cues.

## CRediT authorship contribution statement

**V. Loison:** Writing – review & editing, Visualization, Software, Formal analysis, Data curation, Writing – original draft, Investigation, Validation, Methodology. **Y. Voskobiynyk:** Writing – review & editing, Validation, Resources, Data curation, Writing – original draft, Methodology. **B. Lindquist:** Writing – original draft, Validation, Resources, Investigation, Data curation. **D. Necula:** Writing – original draft, Validation, Resources, Investigation, Data curation, Writing – review & editing, Methodology. **D. Longrois:** Writing – review & editing, Visualization, Supervision. **J. Paz:** Writing – review & editing, Validation, Supervision, Project administration, Methodology, Investigation, Funding acquisition, Conceptualization. **D. Holcman:** Writing – review & editing, Writing – original draft, Validation, Supervision, Project administration, Investigation, Funding acquisition, Formal analysis, Conceptualization, Validation, Methodology.

## Declaration of competing interest

The Authors declare no competing financial interests.

## Data Availability

Data will be made available on request.

## Appendix A Supplementary data

MMC S1 Supplementary material presents complement mathematical analysis and figures.
