# Supplementary material for: Mapping general anesthesia states based on electro-encephalogram transition phases
Source: Neuroimage. 2024 Jan;285:120498. doi: 10.1016/j.neuroimage.2023.120498 (PMC10792552; doi:10.1016/j.neuroimage.2023.120498)
Supplement: MMC S1 — Supplementary material presents complement mathematical analysis and figures. [file mmc1.pdf]

Supplementary information

Mapping general anesthesia states based on electro-encephalogram transition phases

V. Loison[1], Y. Voskobiynyk *[2, 3], B. Lindquist *[2, 3], D. Necula *[2, 3], D. Longrois[4], J. Paz[2, 3], and D. Holcman[1, 5]

[1]Group of Data Modeling and Computational Biology, Institut de Biologie (IBENS), École Normale Supérieure CNRS, Université PSL Paris, France.
[2]Gladstone Institutes
[3]University of California, San Francisco, USA
[4]Département d'Anesthésie-Réanimation, Hôpital Bichat-Claude Bernard, Assistance Publique-Hôpitaux de Paris, Paris, France.
[5]DAMPT, University of Cambridge and Churchill College, CB30DS, Cambridge, UK

December 8, 2023

1 Extended results

1.1 Band behavior during emergence

Although we identified a deterministic and ordered chain of EEG events during induction and maintenance of anesthesia, this chain did not extend to recovery. In particular, we found that the θ and δ activity did not have a characteristic and systematic evolution during emergence. This is illustrated in the average plots of the δ- and θ- relative powers in Figure S7A, where emergence starts at time 25 minutes. For the incremented and 1.5% protocols (left and center columns), during emergence, the relative delta power (green plot) increases to a value higher than the baseline value, while the relative theta power increases very softly (yellow plot). However, the constant 1% plot (right column) shows the opposite behavior: the relative delta power stays very low, while the relative theta power increases drastically.

*Contributed equally.

1.2 1/f-parameters have characteristic behavior

We separated the EEG power spectral density into two components: the oscillatory component and the 1/f-component, with the form $\frac{a_t}{c_t+f^{p_t}}$ where a_t, p_t, and c_t are estimated on sliding time windows (Methods section 4.6, Fig. S7). The 1/f-component exponent p_t had the same behavior during baseline and induction across protocols (Fig. S7B). It started at a relatively stable value between 1 and 1.4, before increasing rapidly after GA began. p_t eventually stopped increasing, and immediately decreased. The maximum value reached by p_t was very close across protocols: 2.07, 2.09, and 2.02 for the incremented, 1.5%, and 1% protocol respectively. However, the duration between start of GA and maximum p_t varied across protocols: 12 minutes, 6 minutes 30 seconds, and 7 minutes for the incremented, 1.5%, and 1% protocol, respectively. The decrease slope of p_t varied across protocols. a_t and c_t behaved globally the same as p_t (Fig. S7C-D).

Moreover, the dynamics of p_t and the relative δ-power P_δ were weakly correlated (0.61 $\pm$ 0.22 Pearson correlation, see Supplementary section 1.2). We found that the 1/f exponent p_t was weakly linked to the δ relative power P_δ (0.61 $\pm$ 0.22 Pearson correlation, 0.58 $\pm$ 0.21 Spearman correlation, see Fig. S7F). On average, the correlation between p_t and P_δ was higher for the incremented protocol and the 1.5% protocol than for the 1% protocol.

1.3 Correlation of 1/-f and oscillatory EEG components

To study the independence of the two components of the spectral decomposition, we computed the correlation of the energy estimated using the area under the 1/f and the oscillatory components. For the latter, we summed the area under the θ and δ Gaussians. We found an insignificant correlation (0.10 $\pm$ 0.2 for the incremented Inj_{Iso} protocol, 0.11 $\pm$ 0.17 for the Inj_{Iso} = 1.5% protocol, and -0.24 $\pm$ 0.2 for the Inj_{Iso} = 1% protocol), with an overall -0.002 $\pm$ 0.25 (Fig. S5F). The present analysis suggests that the two components should be studied separately and bring complementary information.

1.4 Correlation between IES and γ-rebound parameters

In Results section 2.4, we uncovered a phenomenon happening in the EEG γ frequency range. We found that the area under the $\gamma-$power was correlated to the total time spent in IES (Δ_{IES}), which we called cumulative IES duration. We now detail additional $\gamma-$rebound parameters and the possible correlation with IES duration occurring during GA.
We define the γ-rebound duration (Δ_γ) as the time difference between the end of the last γ spindle and the beginning of the first γ spindle during recovery. When we computed Δ_γ in recordings, we did not observe a statistical difference between the anesthesia protocols (Fig. S8A). We next explored whether the γ-rebound durations could be correlated with Δ_{IES}. To address this question, we plotted the points (Δ_{IES}, Δ_γ) for each recording containing a γ-rebound. A linear regression $y = ax + b$ (Fig. S8B) led to $a = 0.58$ and $b = 3.69$ with a determination coefficient $R^2 = 0.2$, revealing no obvious correlation between the γ-rebound duration and IES duration. We note that in 8/23 recordings, the γ-rebound extended beyond

the end of the recording, preventing an accurate estimation of Δ_γ.
To further study the possible correlation between the γ-rebound and Δ_{IES}, we now introduce how the γ-power $p_\gamma(k, t_1, t_2)$ is computed for recording k on the time interval $[t_1,\, t_2]$:

$$p_\gamma(k, t_1, t_2) = \frac{1}{t_2 - t_1} \int_{t_1}^{t_2} S^2_{k,\gamma}(t)\mathrm{d}t, \tag{1}$$

where $S_{k,\gamma}$ is the EEG signal of recording k bandpass filtered in $50-70$Hz, which is the frequency domain activated during γ rebounds. We recall the discretized version:

$$p_\gamma(k, t_1, t_2) = \frac{1}{(t_2 - t_1) f_S} \sum_{t=t_1}^{t_2} S^2_{k,\gamma}(t), \tag{2}$$

where f_S is the sampling frequency. We now introduce the recovery power p_γ^{recov} during the recovery phase for the gamma band as the power computed for $S_\gamma(t)$ between the end (time $\tau_{\text{Iso}}^{\text{stop}}(k)$) of anesthesia and the end (time $\tau_{\text{rec}}^{\text{stop}}(k)$)) of recording k as

$$p_\gamma^{\text{recov}}(k) = p_\gamma(k, \tau_{\text{Iso}}^{\text{stop}}(k), \tau_{\text{rec}}^{\text{stop}}(k)). \tag{3}$$

To further investigate the possible correlation between the gamma recovery power $p_\gamma^{\text{recov}}(k)$ and $\Delta_{\text{IES}}(k)$, (Fig. S8C), we performed a linear regression $y = ax + b$ and found $a = 32.4$, $b = 84.5$ and $R^2 = 0.5$, confirming a weak linear dependency.
Finally, we define the normalized γ-recovery power between the end of anesthesia and the end of recording as

$$p_\gamma^{\text{Nrecov}}(k) = \frac{p_\gamma^{\text{recov}}(k)}{p_\gamma(k, \tau^0(k), \tau_{\text{Iso}}^{\text{start}}(k))}, \tag{4}$$

where $\tau^0(k)$ is the beginning time of the EEG recording k. We performed a linear regression $y = ax + b$ (Fig. S8D) leading to $a = 0.17$, $b = 0.81$, and $R^2 = 0.2$, suggesting no correlation.
To conclude, we found no statistical correlation between the IES duration and $\gamma-$rebound durations, or IES duration and the $\gamma-$rebound energy, normalized or not.

1.5 EEG γ-rebound is not EMG contamination

During the γ-rebound described in Results section 2.4, EEG γ bursts were synchronized with EMG bursts (Fig. S10A-B). But spectral decomposition of these signals showed key differences (Fig. S10C-D). The EEG power spectrum was much more powerful, with power going up to 200 μV as opposed to 20 μV for the EMG. Moreover, during EEG bursts, the γ activity was in a narrow frequency domain, around 60 Hz. On the contrary, in EMG bursts, the entire $50-100$ Hz range was active. Therefore, EMG contamination was not the cause of the high-power activity of the γ-rebound EEG. However, because of their synchronicity, we hypothesize that EEG and EMG bursts are linked, possibly caused by a common phenomenon that has yet to be unveiled.

1.6 δ disappearance predicts the emergence of suppressions several minutes in advance

We investigated the dynamics of the δ- and θ- rhythms and their roles in predicting BS appearance. Interestingly, we found that the δ-rhythm disappearance could anticipate the appearance of BS (Fig.S9).

To obtain a predictive estimate of the arrival of BS, we computed the normalized power P_δ of a sub-delta band in the range 2−4 Hz (see eq. 7). We found that when P_δ leaves for the first time a region where its value exceeds a threshold $T_\delta = 0.15$, BS appear in average with a delay of few minutes (Fig. S9A), in 81% of the cases. Interestingly, this delay was, on average, 4.5 minutes for the 1.5% protocol, and 1.9 min for the incremented protocol (Fig. S9B).

1.7 Repeated isoflurane exposure does not induce group differences in response to anesthesia

We noticed that one individual mouse could respond differently to anesthesia when anesthetized several times. We however found no evidence that this variability was a consequence of repeated exposure to isoflurane. For instance, two mice received the constant 1% protocol three times in a row. Interestingly, the intra-individual response varied greatly. While the first mouse had a cumulative IES duration of 0 min, 0 min and then 1.32 min (suggesting that exposure to isoflurane increases isoflurane sensitivity), the second mouse had a cumulative IES duration of 2.4 min, 0.85 min, and then 1.5 min (suggesting that isoflurane exposure does not have a strong impact on isoflurane sensitivity). We therefore found no general trend as to whether isoflurane exposure history increases or decreases isoflurane sensitivity over time. We further evaluated this question by comparing the cumulative IES duration of two groups of recordings undergoing the incremented protocol. Indeed, the cumulative IES duration can be used as a quantification of sensitivity to anesthesia. In the first group, the mice were naive, they had not been anesthetized before (except during surgical implantation, weeks prior to the experiment) (n=4, mean cumulative IES duration of 4.7 $\pm$ 2.3 min). In the second group, the mice had recently been anesthetized using the constant 1.5% protocol (n=4, mean cumulative IES duration of 2.8 $\pm$ 2.1 min). The Mann-Whitney U rank two-sided test returned a p-value of 0.47, meaning we cannot reject its null hypothesis, which is that the two groups have the same underlying distribution. We conclude that in our recordings, previous isoflurane exposure does not predict the response to isoflurane.

2 Extended methods

2.1 Choice of the threshold value to segment IES

IES detection relies on the choice of a threshold value T_{IES}. Choosing this threshold depends on the amplitude of the EEG signal, which can vary with the type and position of the electrodes, and with inter-individual variability [1]. We describe here how we selected this threshold and adapted the value for each recording. We express the threshold for IES as

$$T_{\text{IES}} = r_{\text{IES}} \text{RMS}_{\text{EEG}}, \tag{5}$$

where RMS_{EEG} is the Root Mean Square (RMS) of the EEG signal computed over the entire duration of each recording and the parameter r_{IES} has to be determined. If r_{IES} is too small, then IES epochs are not correctly detected (Fig. S1A). If r_{IES} is a relevant value, then IES epochs are correctly detected (Fig. S1B). And if r_{IES} is too big, then non-IES epochs are wrongly detected as IES (Fig. S1C). To determine r_{IES}, we first varied r_{IES} between 0 and 1.5 , and for each r_{IES} value, we ran the IES detection algorithm (Methods) during the entire duration of each recording. For each r_{IES} and each recording k, we computed the total time $\widehat{\Delta}^k_{\text{IES}}$ of detected IES that we plotted versus r_{IES} (Fig. S2A1-A2) after we subdivided the recordings into two subgroups:

1. G_1 with IES ($n_1 = 26$),
2. G_2 with no IES ($n_2 = 4$).

The division in G_1 and G_2 was done by visually inspecting the data. The average curves for each subgroup are defined by

$$\widehat{\Delta}_1(r_{\text{IES}}) = \frac{1}{n_1} \sum_{k \in G_1} \widehat{\Delta}^k_{\text{IES}}(r_{\text{IES}}) \tag{6}$$

$$\widehat{\Delta}_2(r_{\text{IES}}) = \frac{1}{n_2} \sum_{k \in G_2} \widehat{\Delta}^k_{\text{IES}}(r_{\text{IES}}), \tag{7}$$

where n_1 (resp. n_2) is the number of recordings in G_1 (resp. G_2), and the relative threshold values r_{IES} were between 0 and 1.5, with an increment of 0.05. The result is plotted with the standard deviation in Fig. S2B1-B2. For mice in G_1, the average curve $\widehat{\Delta}_1(r_{\text{IES}})$ follows a fast increase, followed by a region with a much smaller slope value that we refer to as plateau Pl_{G1} (red in Fig. S2B1). The curve ends with another fast increase. In contrast, the average curve $\widehat{\Delta}_2(r_{\text{IES}})$ stays equal to zero, before rapidly increasing (Fig. S2B2).
We then approximated the first derivative of the average curve $\widehat{\Delta}_1$:

$$\widehat{\Delta}'(r'_n) = \frac{\widehat{\Delta}_{\text{IES}}(r_{n+1}) - \widehat{\Delta}_{\text{IES}}(r_n)}{r_{n+1} - r_n}, \tag{8}$$

with $r'_n = \frac{r_n + r_{n+1}}{2}$.
The plateau Pl_{G1} is visible as a well in the first derivative of Δ_1 (Fig. S2C) and provides an admissible range for the relative threshold r_{IES}, i.e. a set of values with which IES detection is satisfactory. To minimize false positives (IES wrongly detected), we chose the relative threshold at the left bound of the plateau, $r^*_{\text{IES}} = 0.7$. We note that with this value, no IES are wrongfully detected in group G2 (Fig. S2B2).

2.2 Influence of the correcting factor c_t on the 1/f-decomposition

In Methods sections 4.5.1 and 4.5.2, we decomposed the power spectrum into 1/f and oscillatory components on sliding time windows. In this section, we discuss the influence of the parameter c_t on the $1/f$-component estimation. For that purpose, we compared the fits $y_1(f) = \frac{a}{c+f^p}$ and $y_2(f) = \frac{a}{f^p}$ in their capacity to approximate the $1/f$-component in the frequency domain. The parameter estimation for a, c and p for y_1 and a, p for y_2 is described in the Methods section 4.5.1. These fits are performed for all recordings in our dataset and computed over successive time windows with a width of 60 seconds and an overlap of 30 seconds (Fig.S3).
We now describe how we evaluate the local error made by each fitted function on a sliding time window: The 1/f-component of the EEG signal $\tilde{y}^k_{t,w}$ is estimated from the time window $W_w(t)$ of recording k by parameterizing y_1 (resp. y_2) $\tilde{y}^k_{t,w}$, resulting in $y^k_{1,t,w}$ and $y^k_{2,t,w}$ respectively. The local normalized error made by y_1 on time window $W_w(t)$ of recording k is defined by

$$\text{Err}(\tilde{y}, y_1, t, w, k) = \frac{\int_0^\infty (\tilde{y}^k_{t,w}(f) - y^k_{1,t,w}(f))^2 \text{d}f}{\int_0^\infty \tilde{y}^k_{t,w}{}^2 \text{d}f}, \tag{9}$$

and the local normalized error made by y_2 on $W_w(t)$ of recording k is:

$$\text{Err}(\tilde{y}, y_2, t, w, k) = \frac{\int_0^\infty (\tilde{y}^k_{t,w}(f) - y^k_{2,t,w}(f))^2 \text{d}f}{\int_0^\infty \tilde{y}^k_{t,w}{}^2 \text{d}f}. \tag{10}$$

The discretized version of the local normalized error is given by:

$$\text{Err}(\tilde{y}, y_j, t, w, k) = \frac{\sum\limits_{l=\lfloor \frac{m}{\epsilon} \rfloor}^{\lfloor \frac{M}{\epsilon} \rfloor} (\tilde{y}^k_{t,w}(\epsilon l) - y^k_{j,t,w}(\epsilon l))^2}{\sum\limits_{l=\lfloor \frac{m}{\epsilon} \rfloor}^{\lfloor \frac{M}{\epsilon} \rfloor} \tilde{y}^k_{t,w}(\epsilon l)^2}, \tag{11}$$

where $j \in \{1, 2\}$, M (resp. m) is the upper (resp. lower) bound of the frequency range on which we extracted the $1/f$- component, and ϵ is the frequency increment. Here, $m = 0.2$ Hz, $M = 15$ Hz, and $\epsilon = 0.1$ Hz. The local normalized errors made by y_1 and y_2 along one recording

(Fig. S4) are used to estimate the final errors (eq. 11) over all fitted time windows in the entire dataset: it is computed by

$$\mathrm{Err}(\tilde{y}, y_j) = \frac{1}{N}\sum_{k=1}^{N}\frac{1}{N_k}\sum_{m=0}^{N_k-1}\mathrm{Err}(\tilde{y}, y_j, wm + \frac{w}{2}, w, k), \tag{12}$$

where $j \in \{1, 2\}$, N is the number of recordings in the dataset, N_k is the number of sliding time windows in recording k, w is the width of the sliding time windows. Here, $N = 30$ and $w = 1$ minute. We found the average normal errors (computed on all our recordings) are given by

$$\mathrm{Err}(\tilde{y}, y_1) = 0.068 \pm 0.02$$
$$\mathrm{Err}(\tilde{y}, y_2) = 1.038 \pm 0.44.$$

To conclude, according to this error criteria, the y_1 fit performs better than the y_2 fit with a factor of 15, thus confirming the relevance of the correction term c_t.

3 Supplementary figures

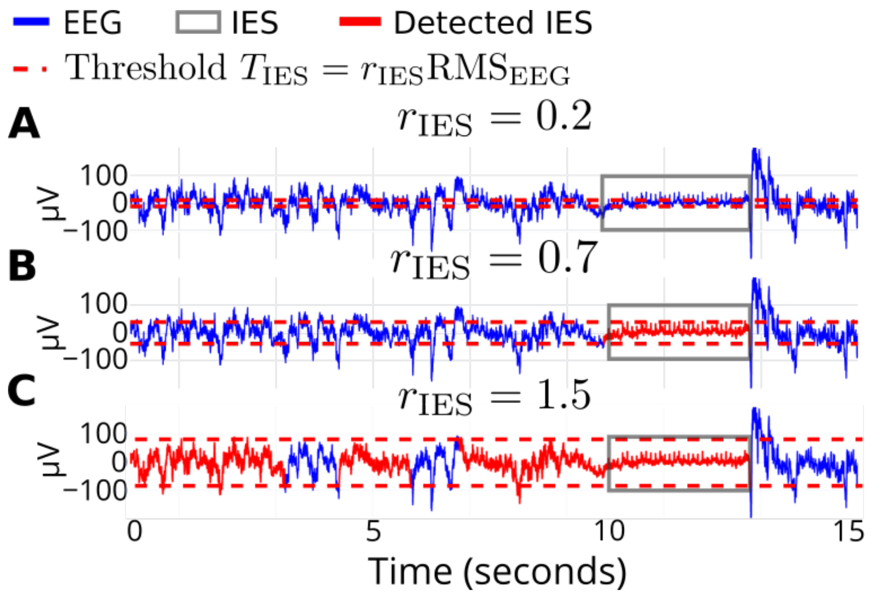

Figure S1: **IES detection depends greatly on the threshold.** EEG showing an IES in the range 10 − 13 seconds. The IES is detected using the method described in section 2.1 for $r_{\mathrm{IES}} = 0.2$ **(A)**, $r_{\mathrm{IES}} = 0.7$ **(B)**, $r_{\mathrm{IES}} = 1.5$ **(C)**.

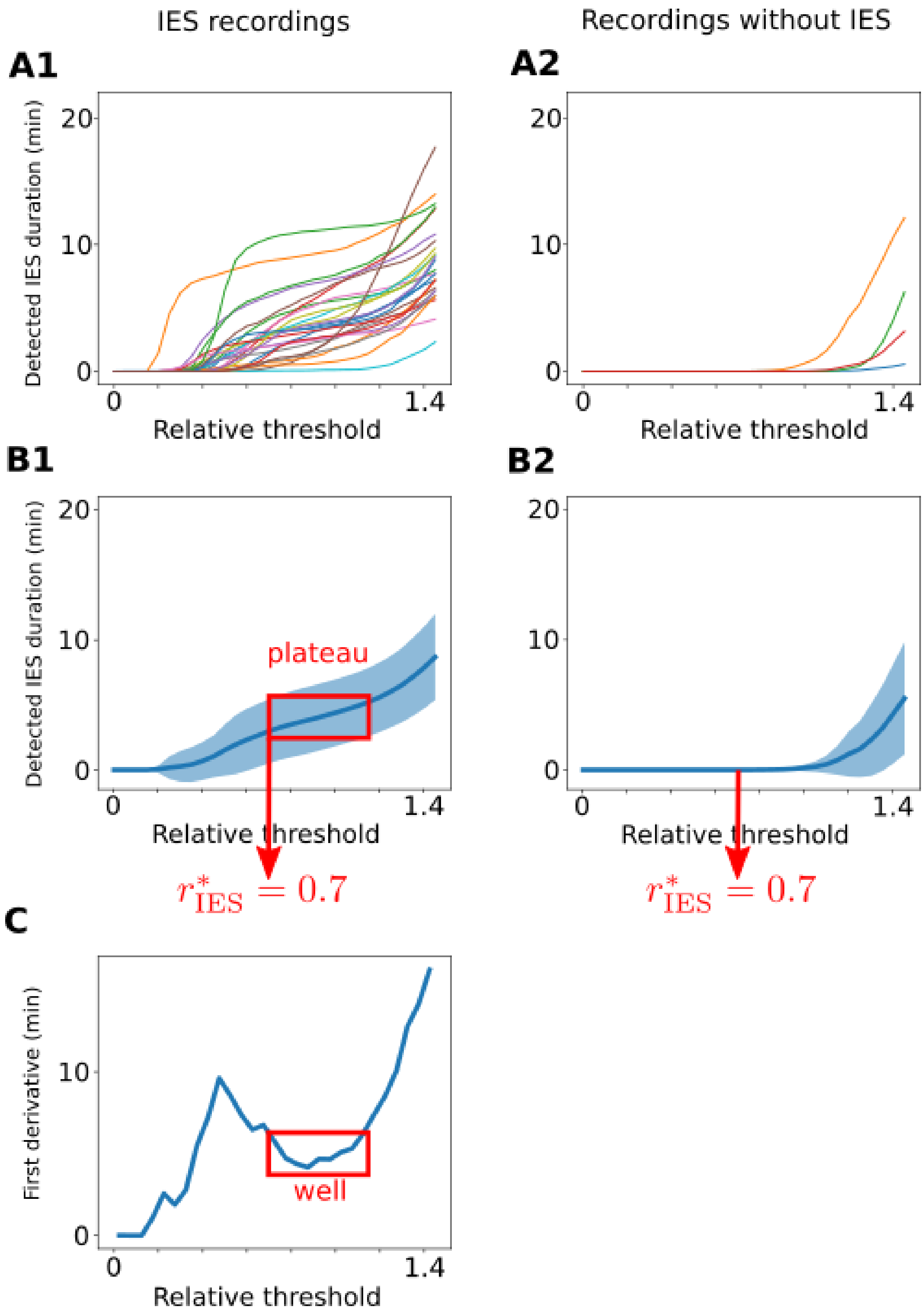

Figure S2: **Choice of relative threshold r_{IES} for IES detections.** Detected IE duration $\hat{\Delta}^k_{\mathrm{IES}}$ versus relative threshold r_{IES} for recordings with IES **(A1)**, and recordings without IES **(A2)**. **(B1)** Average curve (full) with the standard deviation (blue area) of the curves presented in **(A1)**. **(B2)** average with the standard deviation of curves in **(A2)**. **(C)** Derivative of the mean detected IE duration $\hat{\Delta}^1_{\mathrm{IES}}$. The admissible range for the relative threshold r_{IES} is the slowest increase region in **(B1)**, associated with a well **(C)**. We chose the value $r^*_{\mathrm{IES}} = 0.7$ (red), located at the left of the well.

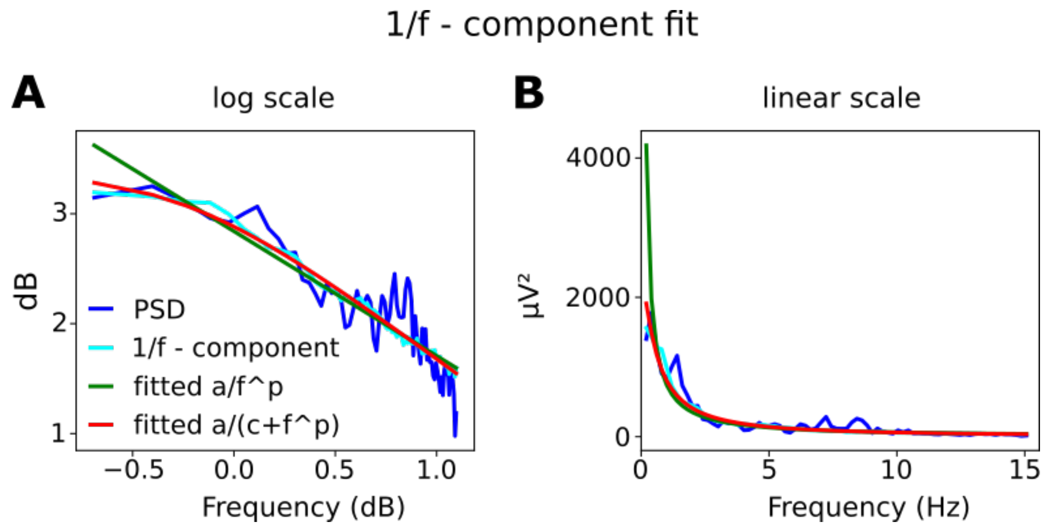

Figure S3: **1/f-component in the frequency space. (A)** The power spectral density (dark blue) is fitted by an estimate $\tilde{(y)}$ computed over one minute (IRASA $1/f$ component extraction in light blue), $y_1 = \frac{a}{c+f^p}$(red) and $y_2 = \frac{a}{f^p}.\ \tilde{(y)}$ (green). The parameters $a, c,$ and p are estimated in the Methods section. **(B)** linear scale.

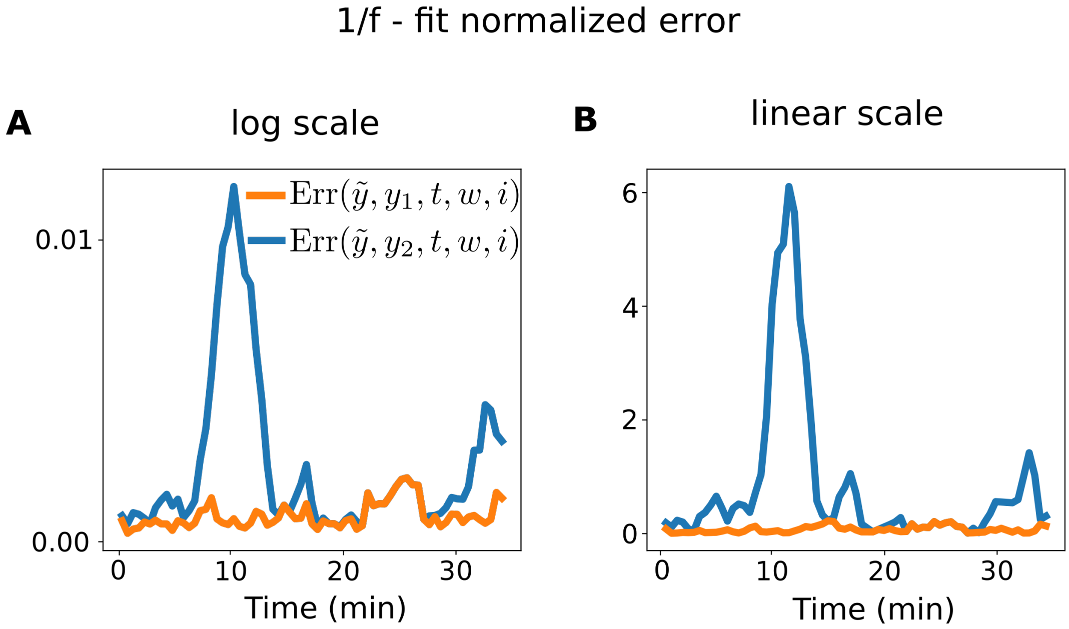

Figure S4: **Local errors associated with $y_1 = \frac{a}{c+f^p}$ and $y_2 = \frac{a}{f^p}$ decomposition, computed over the EEG recording. (A)** log and **(B)** linear scale.

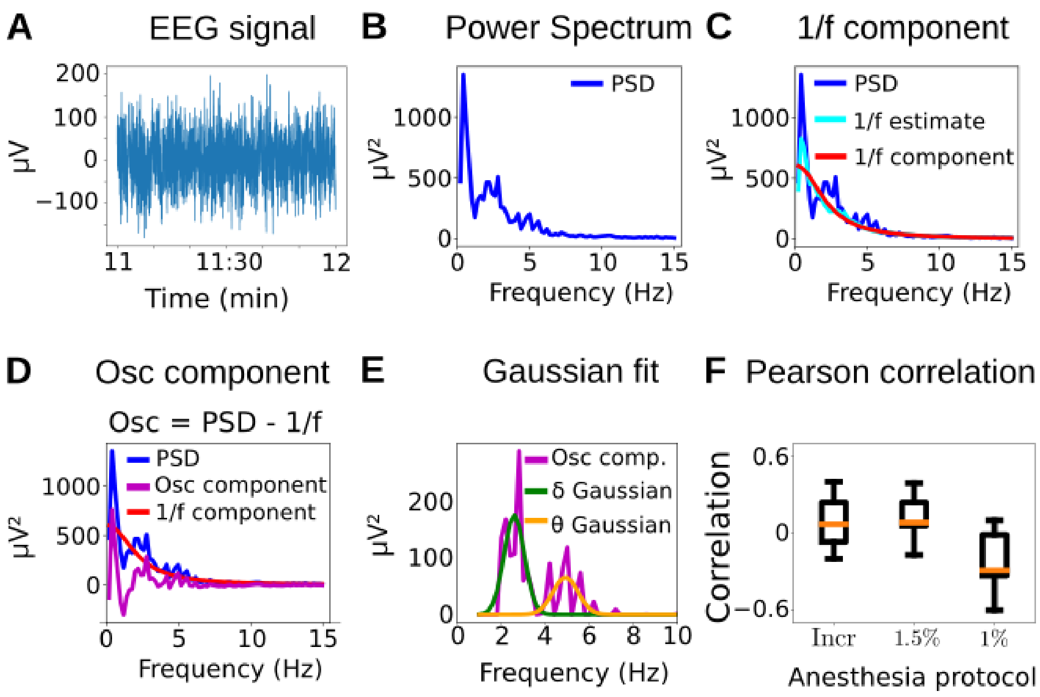

Figure S5: **Estimation of oscillatory and fractal parameters on a time window. (A)** Input window of the signal. **(B)** Power Spectral Density of the input signal. **(C)** Estimation of the fractal component and the fractal parameters a_t, p_t, c_t. **(D)** Estimation of the oscillatory component. **(E)** Decomposition of the oscillatory component in Gaussians and parameter extraction of the θ- and δ- rhythms. **(F)** Correlation between the areas under the fractal and oscillatory components.

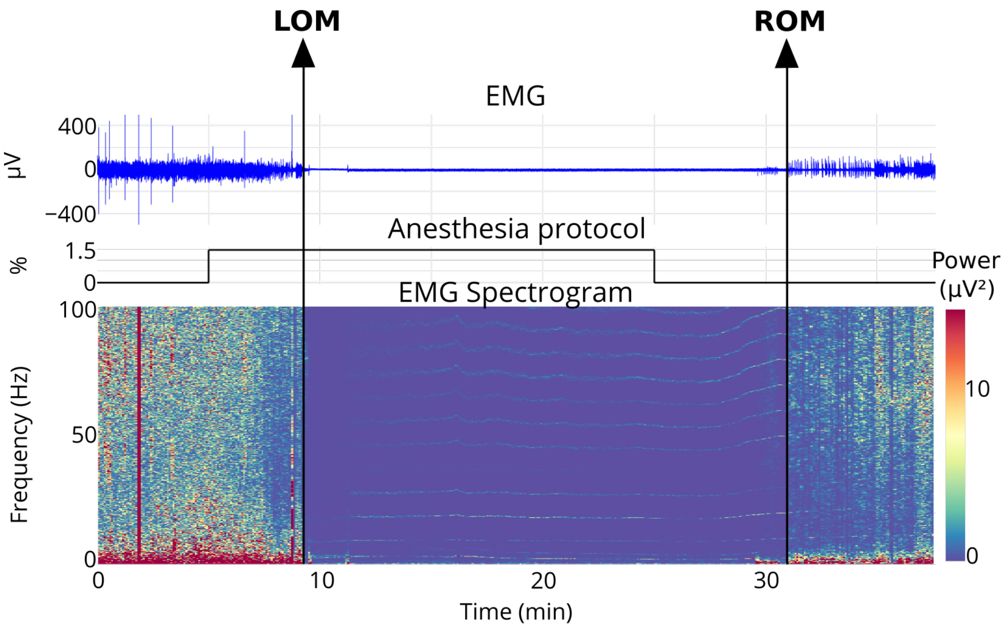

Figure S6: **EMG and associated spectrogram over an entire recording**. Loss of Movement (LOM), indicated by the first vertical black arrow, and Return of Movement (ROM), indicated by the second black arrow.

	Incr. $\mathrm{Inj_{Iso}}$	$\mathrm{Inj_{Iso}} = 1.5\%$	$\mathrm{Inj_{Iso}} = 1\%$	All	p-value
$\tau_\theta^{\mathrm{decay}} - \tau_{\mathrm{Iso}}^{\mathrm{start}}$	1.3 ± 1.4	0.9 ± 0.7	0.6 ± 0.3	1 ± 1.1	6.6×10^{-6}
$\tau_\delta^{\mathrm{app}} - \tau_\theta^{\mathrm{decay}}$	6.0 ± 1.8	2.4 ± 1.4	3.1 ± 2.4	4 ± 2.5	3.5×10^{-5}
$\tau_\theta^{\mathrm{disp}} - \tau_\delta^{\mathrm{app}}$	6.4 ± 3.8	3.5 ± 1.1	2.6 ± 2.5	4.6 ± 3.4	4.6×10^{-6}
$\tau_\delta^{\mathrm{disp}} - \tau_\theta^{\mathrm{disp}}$	3.4 ± 2.1	4.4 ± 1.9	3.6 ± 5.3	3.8 ± 3.1	8.2×10^{-6}
$\tau^{\mathrm{S}} - \tau_\delta^{\mathrm{disp}}$	1.9 ± 1.8	4.5 ± 3.1	6.0 ± 0.3	3.2 ± 2.7	9.9×10^{-5}

Table 1: **Time difference between key EEG events (mean $\pm$ std in minutes), and p-value of the one-sided Wilcoxon signed-rank test.**

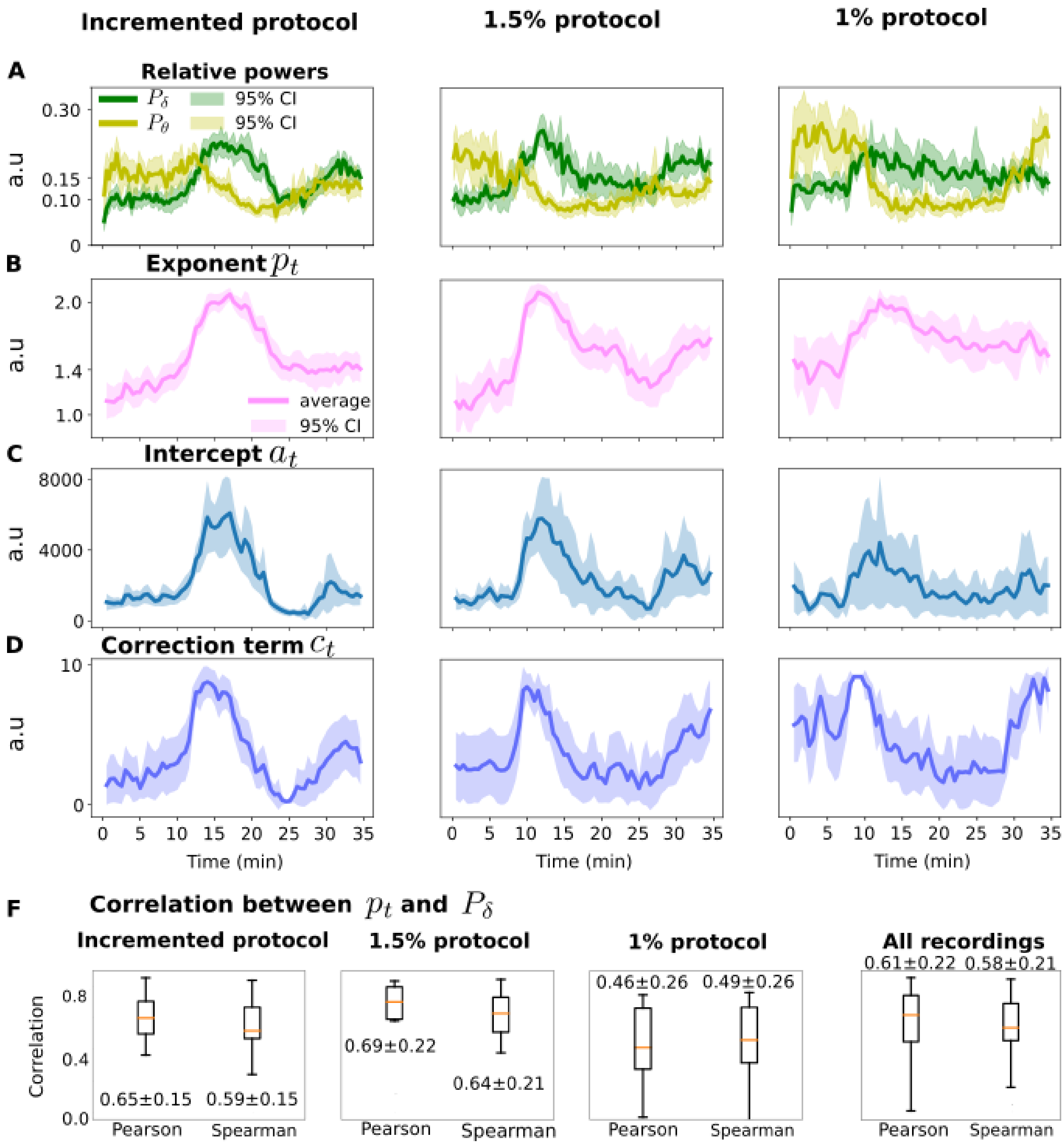

Figure S7: **Trajectories of spectral parameters for anesthesia protocol: relative powers and 1/f- component** $\frac{a_t}{c_t+f^{p_t}}$**.** **(A)** Relative δ-power (dark green) and relative θ-power (yellow). **(B)** exponent p_t, **(C)** intercept a_t and **(D)** correction term c_t. **(E)** Distribution of Pearson and Spearman correlations between p_t and P_δ. Error bands indicate the 95% confidence intervals computed using the t-distribution.

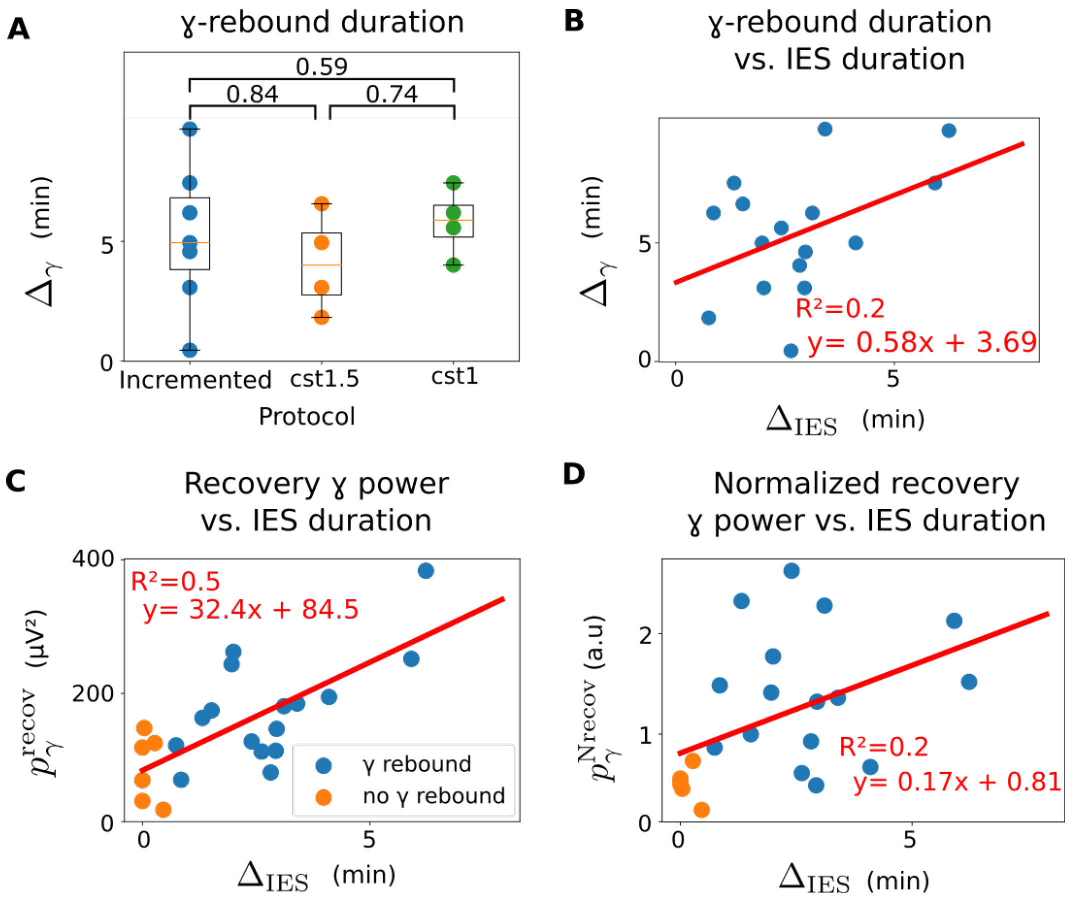

Figure S8: **Statistical parameters associated with the $\gamma-$rebound.** **(A)** The γ-rebound duration is not statistically different among the three anesthesia protocols (two-sided Wilcoxon-rank U test). **(B)** γ-rebound duration is not linearly correlated to IES duration. Recovery γ power **(C)** and normalized recovery γ-power **(D)** are not linearly correlated to IES duration.

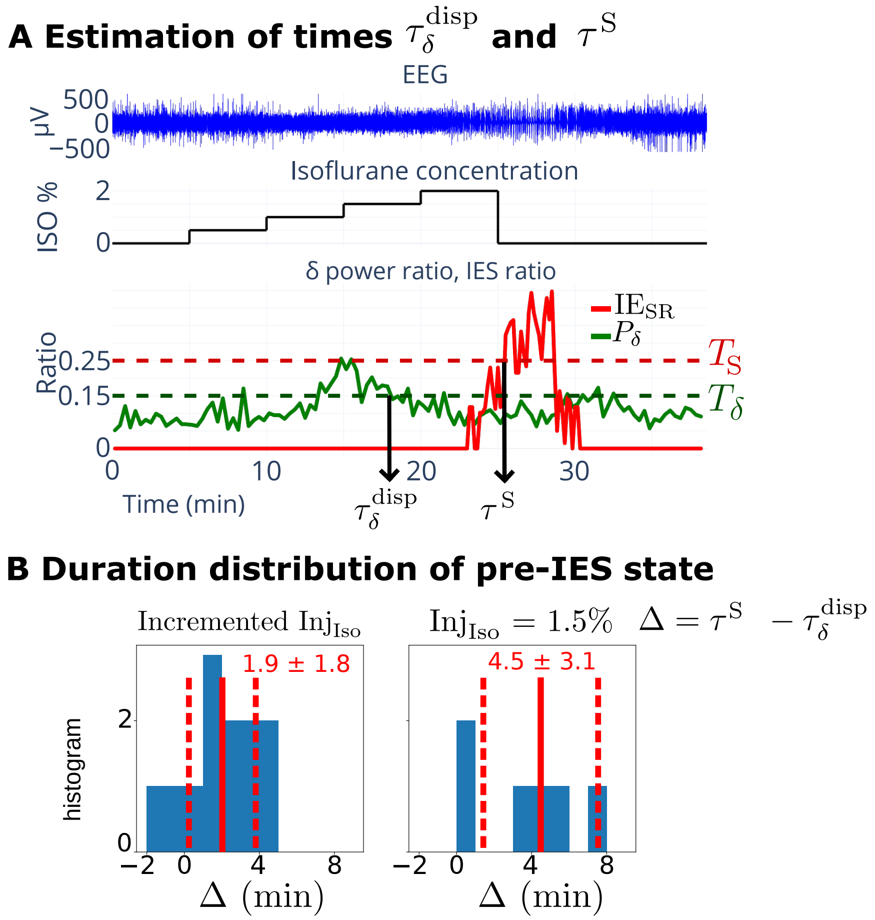

Figure S9: δ**-rhythm disappearance precedes IES by several minutes.** **(A)** Estimation of δ-rhythm disappearance time $\tau_\delta^{\text{disp}}$ and IES time τ^{S}. **(B)** Distribution of the time difference between $\tau_\delta^{\text{disp}}$ and τ^{S} for two protocols.

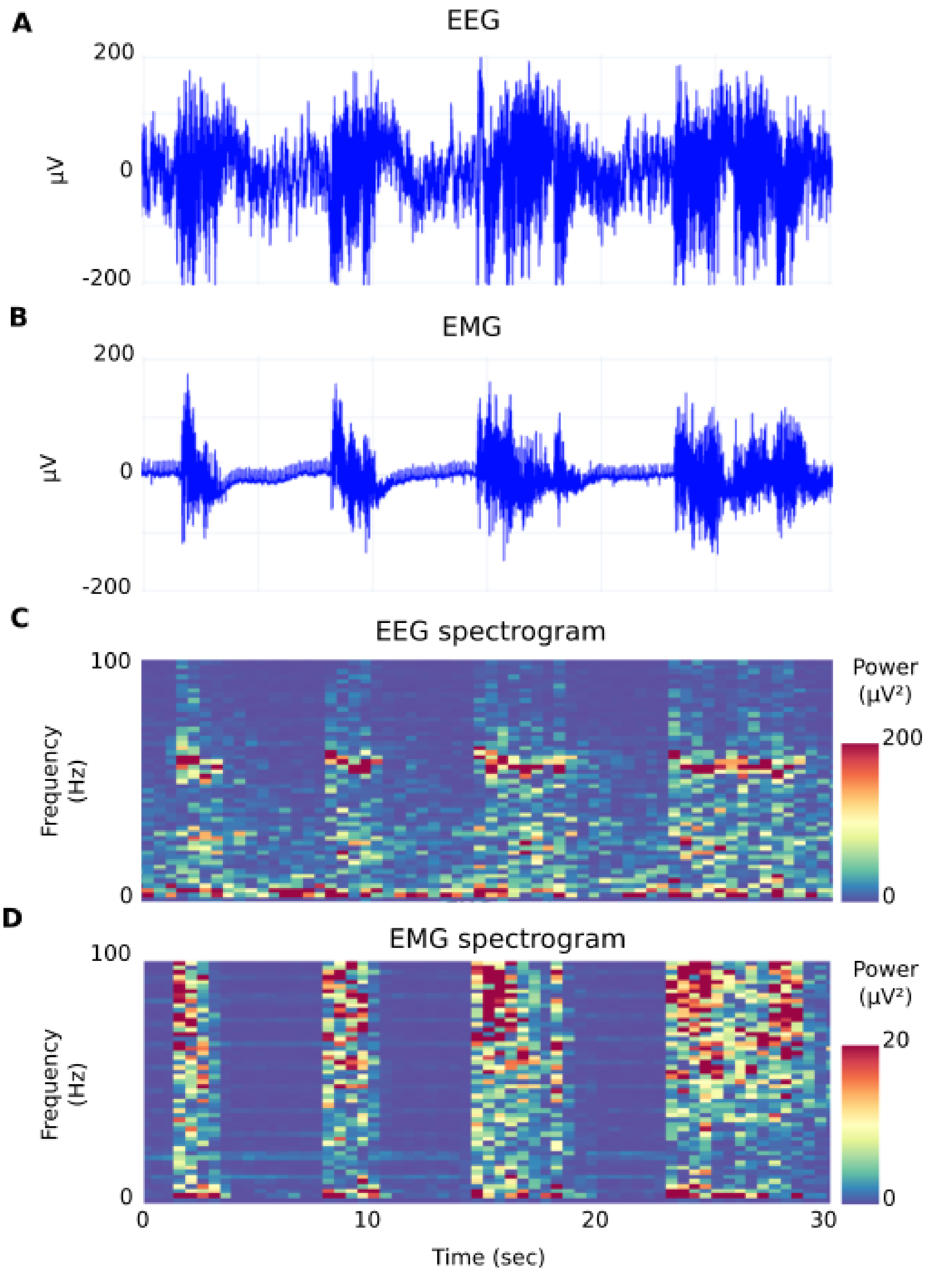

Figure S10: **Magnification of EEG and EMG during a γ-rebound.** **(A)** EEG signal. **(B)** EMG signal. **(C)** EEG spectrogram. **(D)** EMG spectrogram. The EMG spectrogram differs greatly from the EEG spectrogram, showing that the EEG γ-rebound is not EMG contamination.

References

[1] M. X. Cohen, *Analyzing neural time series data: theory and practice.* MIT press, 2014.
